# Icephobicity of Oil-Infused Silicone Elastomer Coatings: Ice Adhesion, Freezing Time, and Room-Temperature Characterization

**DOI:** 10.1021/acsomega.5c01139

**Published:** 2025-04-08

**Authors:** Catherine M. Megregian, Vasileios Koutsos, Anthony Callanan, Jane R. Blackford

**Affiliations:** † School of Engineering, Institute for Materials and Processes, The University of Edinburgh, Sanderson Building, King’s Buildings, Edinburgh EH9 3FB, U.K.; ‡ School of Engineering, Institute for Bioengineering, The University of Edinburgh, Faraday Building, King’s Buildings, Edinburgh EH9 3DW, U.K.

## Abstract

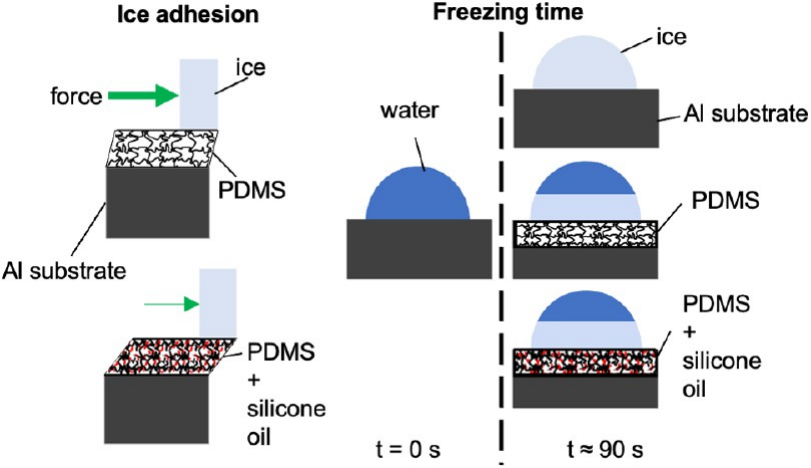

Passive anti-icing coatings are a promising solution to the dangers of ice accumulation on surfaces. We studied plain polydimethylsiloxane (PDMS) and (commercially available) NuSil R-2180 coatings alongside PDMS coatings infused with two molecular weights and percentages of silicone oil. The icephobicity of the coatings was measured via ice adhesion strength and freezing time. 100 repeated deicing cycles were performed, which showed the oil-infused coatings had consistently lower ice adhesion strengths (∼10–20 kPa) than nonoil-infused coatings (∼100 kPa). The nonoil-infused coatings also showed increasing instances of exceptionally high ice adhesion strengths (>650 kPa), reducing the reliability of their icephobicity long-term. Oil infusion did not negatively affect the freezing time of the coatings, and despite decreases in freezing time after 100 deicing cycles, the coatings maintained an improvement compared to uncoated aluminum. Analysis showed adhesion strength is more strongly affected by shear modulus than coating thickness, work of adhesion, or static water contact angle. Wear from the deicing cycles was minimal. Any wear that was present did not significantly affect icephobicity. Oil infusion of elastomer coatings reduces ice accumulation on surfaces and provides a more reliable long-term solution for anti-icing applications.

## Introduction

Ice accumulation on surfaces is a widespread issue, affecting applications such as aerospace, energy, and transportation infrastructure. Accumulation can cause downtime to services, accruing unnecessary labor and energy costs;^1^ disruption of critical energy supply;^2^ more hazardous and expensive transportation;^3^ and severe or fatal accidents. To mitigate these hazards, ice accumulation is actively prevented in a variety of ways. This can include the very mundane scraping of a car windscreen, road salting, or more specialized chemical, mechanical, or thermal removal methods. However, all active methods require repeated intervention, which can be costly, inefficient, and labor-intensive. The energy and material expenditure associated with active deicing is undesirable. The environmental impacts of active deicing are also a concern, such as groundwater contamination from chemical deicing.^4^

Passive anti-icing coatings provide an alternative to active deicing methods and could reduce, or eliminate, the requirement for active deicing, saving time, money, labor, and other resources. They are also preventative rather than reactive, reducing possible damage from ice accumulation. Anti-icing coatings have shown reasonable icephobicity, here defined as the “resistance to ice accumulation”. Icephobicity is commonly measured via two metrics: ice adhesion strength and freezing time.^5−7^ Ice adhesion strength is the peak ice detachment force divided by the contact area. An upper limit of ice adhesion strength for passive anti-icing has been suggested as 100 kPa^8^ but is likely closer to 10–20 kPa.^9−11^ Freezing time is the time taken for a droplet of water to freeze on a surface from deposition. The lower the ice adhesion strength and the greater the freezing time, the better the icephobicity. Together these metrics describe the ability to mitigate ice formation and accumulation.

The development of anti-icing coatings started with hydrophobic surfaces, which utilize low surface energies to repel water and increase the water contact angle on the surface. The most well-known examples are fluorinated hydrocarbons, such as polytetrafluoroethylene (PTFE/Teflon), which has shown an ice adhesion strength of approximately 300 kPa, compared to 1600 kPa on aluminum.^12^ However, alternatives to fluorocarbons are desired for environmental concerns. Due to the limitations of reducing surface energy via functional group alteration of hydrophobic surfaces, and as they only reduce ice adhesion strength to around 100–300 kPa,^13−16^ other coating technologies have since become more popular areas of research.

Other surfaces which have been investigated are superhydrophobic surfaces (SHS) and slippery-liquid-infused porous surfaces (SLIPS). Inspired by natural surfaces, they utilize complex, hierarchical morphology to increase contact angles and reduce contact area.^17^ Both incorporate fluid-filled spaces in the solid surface, which interrupt and limit the liquid–solid contact. The fluid is air in SHS and liquids (usually oils) in SLIPS. The liquid-fluid adhesion is significantly weaker than liquid–solid adhesion. In the case of SHS, this partial wetting of the solid is called Cassie–Baxter wetting.^18^ However, this wetting type can collapse under external pressure^19^ or after damage to the morphology.^20,21^ The subsequent evacuation of the interspersed fluid leads to unfavorable Wenzel wetting and a higher liquid–solid contact area.^22^ There are also fundamental mechanical differences in the detachment of water and ice,^8^ so the commonly made assumption that hydrophobic surfaces are icephobic is inherently flawed.^23^ Some studies on SHS and SLIPS have shown worsened icephobicity^20,24^ and question their viability as icephobic surfaces.^25,26^ This may be a result of different wetting states, with a possible transition from Cassie–Baxter to Wenzel wetting during the freezing process, by the expansion of ice into the textures or the freezing of microdroplets or condensation within the surface features. The specific mechanisms for ice formation and accretion may affect the wetting state too. Furthermore, promotion of ice nucleation has been shown on certain morphologies.^27−30^

To combat the disadvantages of SHS and SLIPS, and focusing on icephobicity rather than hydrophobicity, alternative coatings without engineered surface textures have been investigated. Recent innovations have been made using low-surface energy elastomer coatings, especially those that are infused with miscible oils.^9,31−33^ Some of these materials have been shown to have incredibly low ice adhesion (<20 kPa). The effect of the addition of miscible oil to elastomers is perhaps most notably recorded by Golovin et al.,^9^ who characterized the ice adhesion strength of a large number of coatings. They identified that reducing the cross-linking density and providing free oil chains can greatly reduce ice adhesion. These results have been supported by other studies;^11,31−34^ however, there was no characterization of the freezing time on these surfaces. Investigating the effect of oil addition on the freezing time will give a more comprehensive understanding of the icephobicity of these coatings and allow for better assessment of their viability as real anti-icing solutions.

In this study, we examined seven elastomer coatings. The freezing time and ice adhesion strength were measured on coatings infused with two different molecular weights and percentages of infused silicone oil, as well as noninfused polydimethylsiloxane (PDMS) and a commercial silicone elastomer, NuSil R-2180. Freezing time was measured on the coatings before and after ice adhesion testing to determine the influence of repeated deicing cycles on the surface durability and long-term icephobicity. Icephobicity observations were supplemented with room-temperature characterization: elastic moduli, contact angle, thickness, and optical and scanning electron microscope (SEM) imaging.

## Experimental Section

### Materials

The two-part PDMS kit, Sylgard 184 was used as the base for all the PDMS or PDMS mixture coatings with the surface primer DOWSIL 1200 OS, both purchased from Silmid (Birmingham, UK). Two molecular weights of silicone oil (hydroxy-terminated PDMS) were used: low molecular weight silicone oil (LMWSO) = 6000 and high molecular weight silicone oil (HMWSO) = 28000. The viscosities of the LMWSO and HMWSO are 100 and 1000 cSt, respectively. Both were purchased from Fisher Scientific (Leicestershire, UK), as well as solvents hexane and isopropanol. The two-part elastomer kit NuSil R-2180 and surface primer NuSil SP-270 were purchased from Polymer Systems Technology Ltd. (High Wycombe, UK).

### Coating Mixture Preparation

To prepare the PDMS-based coatings, the Sylgard 184 base was mixed with the curing agent at a ratio of 10:1 (5.00 and 0.50 mL, respectively). The silicone oil was added, if required, at 5.50 mL (50%) or 1.83 mL (25%). NuSil R-2180 is provided in two medium viscosity parts (Part A and Part B) of a proprietary silicone elastomer dispersed in xylene. To prepare the NuSil R-2180 coatings, Part A and Part B were mixed at a ratio of 1:1 (5.00 mL of each). After mixing, the PDMS-based coatings were left for 30 min at room temperature. Details of the coating mixtures are provided in Table 1.

**Table 1:** Coating Mixture Details

coating name	sylgard 184 vol %	oil vol %	oil molecular weight	oven curing schedule^a^
PDMS 80	100	0		120 min@80 °C
PDMS 100	100	0		45 min@100 °C
25% LMWSO	75	25	low	120 min@80 °C
50% LMWSO	50	50	low	120 min@80 °C
25% HMWSO	75	25	high	120 min@80 °C
50% LMWSO	50	50	high	120 min@80 °C
NuSil R-2180				45 min@75 °C + 135 min@150 °C

a All specimens had an initial room-temperature cure of 30 min before heat curing in oven.

### Specimen Fabrication

To fabricate the icephobicity specimens, aluminum slides measuring 75 mm × 25 mm × 1 mm were cleaned with hexanol and isopropanol and then primed with either DOWSIL 1200 OS (PDMS-based coatings) or NuSil SP-270 (NuSil R-2180 coatings). Primers were cured for 30 min at room temperature. Square molds (20 mm × 20 mm) were taped to the slides, into which the coating mixtures were dispensed by syringe. For PDMS-based coatings, the dispensed volume was 0.20 mL; for NuSil R-2180 coatings, the dispensed volume was 0.70 mL. The NuSil R-2180 specimens were vacuum degassed for 30 min. The specimens were then heat cured using the schedules in Table 1. After curing, the molds were removed from the specimens and any meniscus trimmed away. Two specimens were fabricated of each coating type. Thickness of the coatings was approximately 200 μm. Measurement details provided below. To fabricate the elastic modulus test specimens, the mixtures were poured into and cured in aluminum dishes. Three cylindrical specimens (diameter = 4 mm) were punched from each cast film. Film thickness was measured by digital caliper to be 1.5–3.0 mm.

### Room Temperature Characterization

#### Elastic Modulus

The elastic modulus of the coating types was determined via compression testing on the specimens punched from cast films. Measurements were performed with an Instron 3367 Universal Testing Machine (High Wycombe, UK) with a 50 N load cell. Specimens were compressed between platens at a rate of 20% strain/min, up to 50% strain. Elastic modulus was determined from the gradient of the initial linear elastic region of the resulting stress–strain curves. Measurements were performed on three specimens of each coating type.

#### Coating Thickness

Coating thickness of the icephobicity specimens was measured using a Mitutoyo SJ-410 stylus profilometer (Andover, UK). The icephobicity specimens were taped to a foam block and placed on the profilometer stage. Stylus traces were performed along the specimen, starting on the aluminum slide, passing over the coating, and falling back to the aluminum. Probe speed was 0.5 mm/s. The average step height of the coating (thickness) was calculated from the trace. Five measurements were performed on each specimen.

#### Static Contact Angle

**Figure 1:**
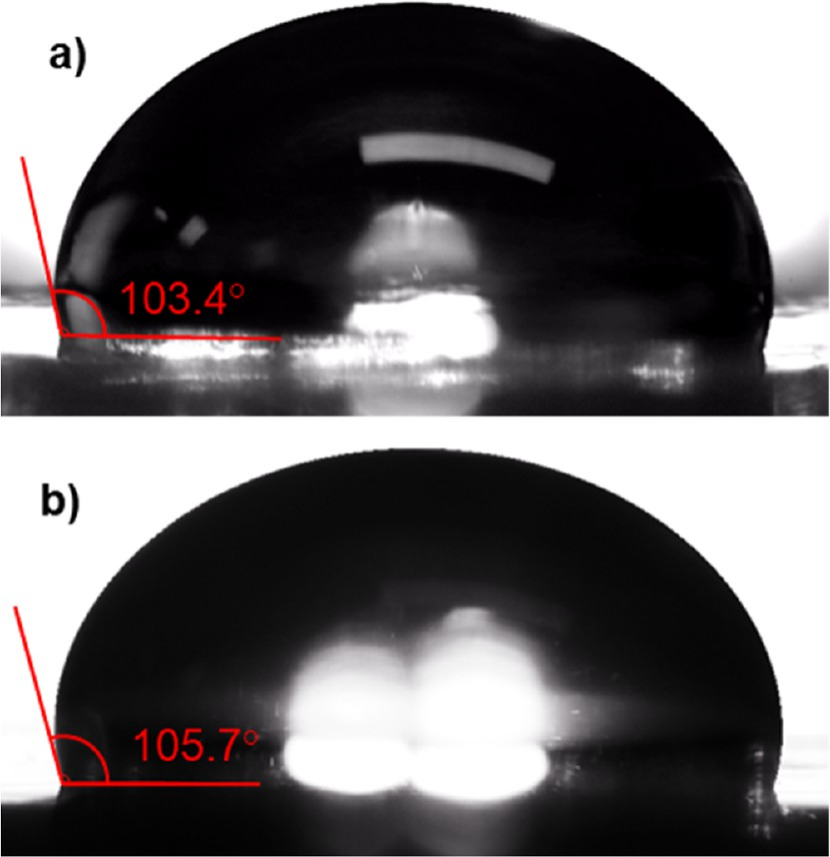
Photographs of water droplets on a 50% LMWSO specimen (a) preadhesion and (b) postadhesion. Contact angles, as measured using the ImageJ software, are denoted on each image.

Static contact angle measurements were performed on the icephobicity specimens before and after adhesion testing. A 0.05 mL droplet of deionized water was placed on the surface of each specimen. The droplet was photographed while backlit. The droplet was removed, and any remaining water was blotted from the surface. Images were processed using ImageJ (IJ 1.46r, Bethesda, MD, USA) contact angle plug-in to measure the contact angle of the droplet. Example images and measured angles are presented in Figure 1. Three droplets were measured on each specimen, and each image was processed thrice. Measurements were performed before and after ice adhesion testing.

#### Optical Microscopy

Optical microscopy was used to visualize the surfaces of the icephobicity specimens with surface oil (25% HMWSO and 50% HMWSO). Images were collected using a Zeiss Axioscope 2 microscope (Cambridge, UK) before and after adhesion testing.

#### Scanning Electron Microscopy

Scanning electron microscopy, performed with a Hitachi TM4000 plus (Maidenhead, UK), was used for visualization of the icephobicity specimens without surface oil. Secondary electron imaging of the as-fabricated coatings was carried out with a 5 kV accelerating voltage under a moderate vacuum. Imaging was performed before and after the adhesion testing regimen.

### Ice Adhesion

**Figure 2:**
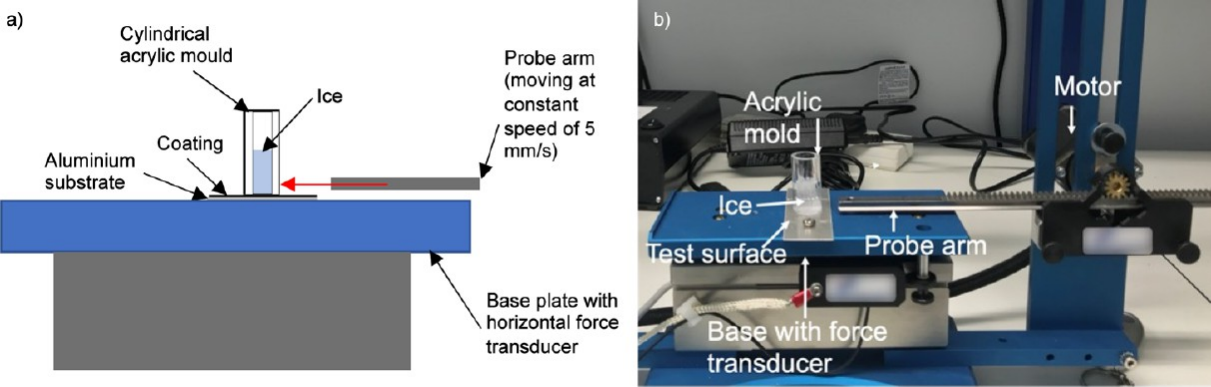
A (a) schematic and (b) photograph of the ice adhesion test setup. A ForceBoard is used to perform a push test on an ice cylinder frozen to the test surface.

Ice adhesion testing was carried out inside a cold laboratory environment, maintained at a temperature of −10 °C ± 1 °C and a relative humidity of 80% ± 10%. Ice adhesion testing was performed using a push test on a ForceBoard (Bastad, Sweden) (Figure 2). A cylindrical acrylic mold was placed on top of the coated specimen, which was bolted to the ForceBoard base plate. The mold had dimensions of 10 mm internal diameter and wall thickness of 1 mm; the height measured 25 mm. Deionized water was allowed to cool to 1 °C in a beaker, and then 1 mL of chilled water was dispensed into the mold by syringe. The water was then left to freeze and cool to −10 °C overnight.

The ice adhesion force was then measured by displacing the ice (frozen in the mold) from the specimen surface and recording the force required for detachment. The ice was displaced by the metal probe arm moving at a constant speed of 5 mm/s, and the horizontal force was measured via transducers in the base plate. The probe height was 1 mm. The adhesion strength, σ_A_, was calculated via
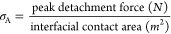
1

Tests were repeated, for a total of 100 tests, over nine months.

### Freezing Time

**Figure 3:**
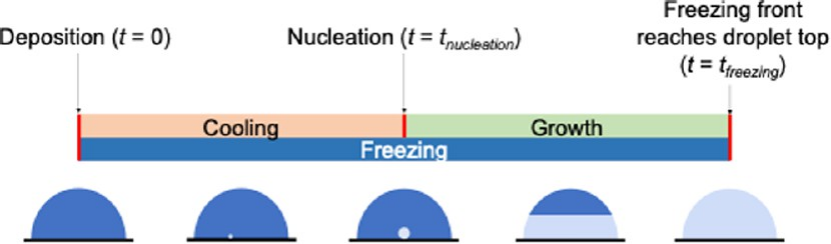
Freezing time, *t*_freezing_, is made up of cooling, nucleation, and growth of ice in a water droplet once it has been deposited on a surface. The cooling stage is marked by the formation of unstable ice embryos before a critical diameter is reached, and the ice nucleus is formed.

Freezing time testing was also carried out inside the cold laboratory. The freezing time, *t*_freezing_, is defined as the time from the deposition of a droplet on a subzero surface to the complete freezing of the droplet. Freezing is complete when the freezing front has reached the top of the droplet and is determined visually. A graphical representation of this is presented in Figure 3.

**Figure 4:**
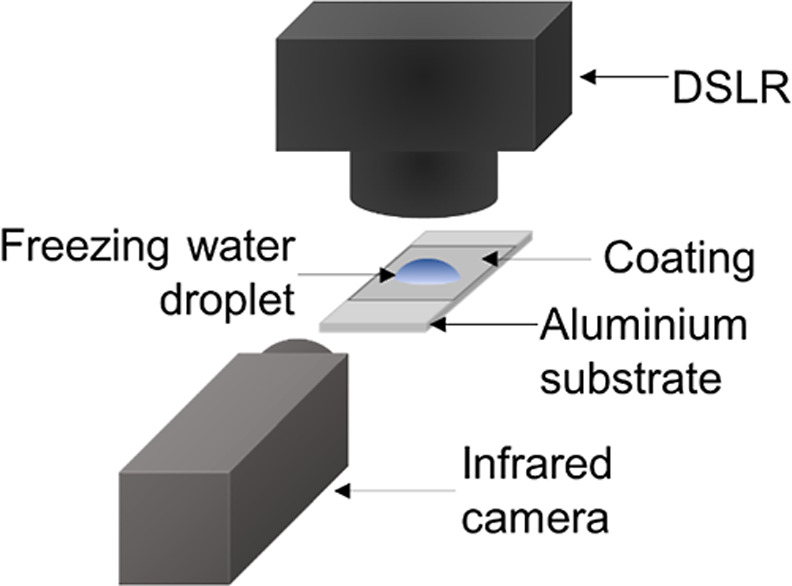
Schematic showing the freezing time test setup with both DSLR and infrared cameras.

The icephobicity specimens were placed on a flat surface, directly below a digital single-lens reflex (DSLR) camera and facing a FLIR A655sc infrared camera (Kent, UK) (Figure 4). After thermal equilibration of the specimens, deionized water was chilled to 8 °C. A 0.05 mL droplet of water was deposited onto the surface and left to freeze. The process was video recorded, and the time from deposition to freezing was determined.

**Figure 5:**
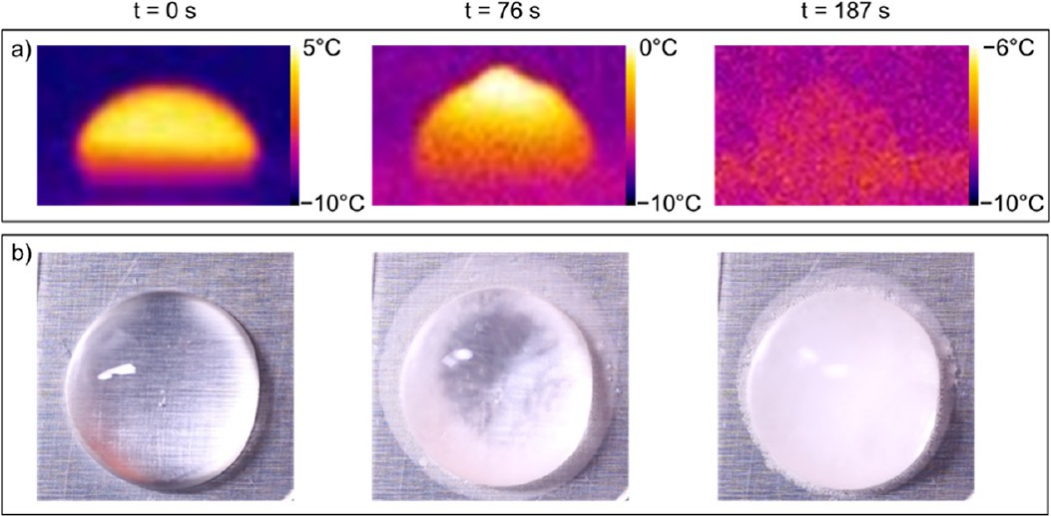
Infrared (a) and camera (b) images of a droplet freezing at 0, 76, and 187 s. Droplet volume is 0.05 mL with a diameter of approximately 6 mm. Condensation is visible around the edge of the droplet at 76 and 187 s and has frozen in spots.

Example visual indications of complete freezing are presented in Figure 5. A minimum of 8 tests were performed on each specimen, before and after the ice adhesion testing. Infrared imaging demonstrated that thermal equilibrium was reached within 6 min of deposition. Recordings were terminated after 7 min if nucleation had not occurred.

## Results and Discussion

### Room-Temperature Characterization

#### Elastic Modulus

**Figure 6:**
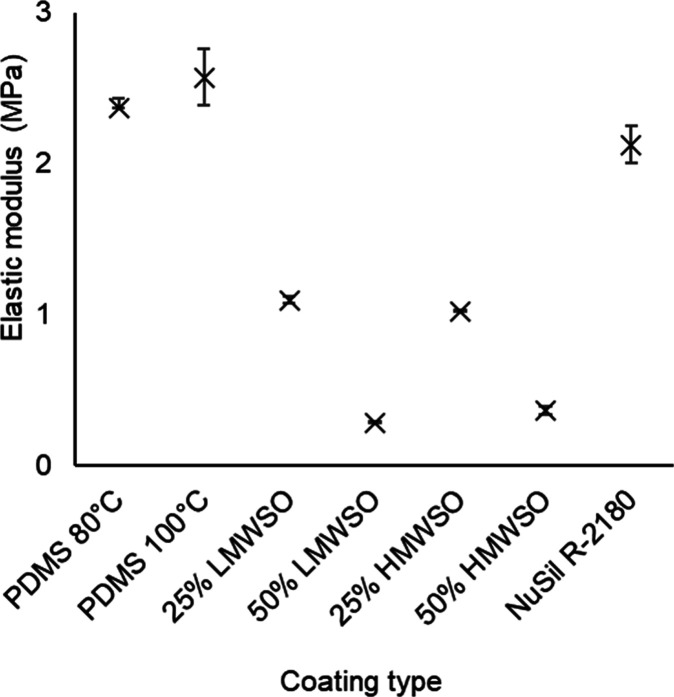
Elastic moduli of the coating types, as measured by compression tests.

The findings from the modulus testing are presented in Figure 6. The average value for the PDMS at 100 °C is 2.57 MPa. The oil-infused coatings all had lower moduli than the nonoil-infused coatings, with a range of 0.29–1.10 MPa. The nonoil-infused coatings have a range of 2.12–2.57 MPa. The nonoil-infused coatings had similar moduli, with the PDMS 80 °C slightly lower than PDMS 100 °C, and the NuSil R-2180 is slightly lower than both PDMS types. The percentage of oil has a much stronger influence on the elastic modulus than the molecular weight of the oil, with similar elastic moduli across the two 25% coatings and the two 50% coatings. The coating with the lowest modulus was the 50% LMWSO coating.

#### Coating Thickness

**Figure 7:**
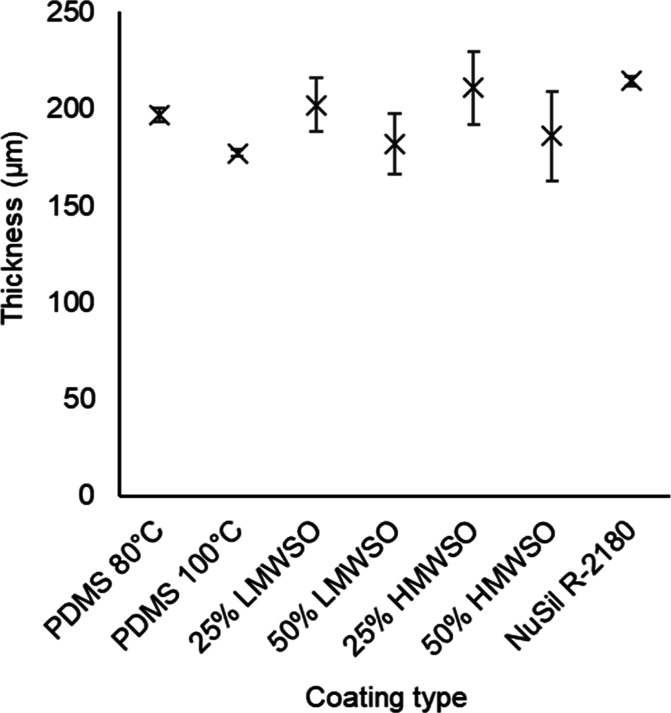
Thicknesses of the coating types. Values are the average of two specimens for each coating type with the spread between the two specimens indicated by range bars. Thickness of each specimen was calculated as the average of 5 evenly spaced profiles.

The thickness measurements are presented in Figure 7. The specimens have a thickness of 200 μm ± 10%. The values presented on the figure are the average for both specimens for each coating type. The range bars provided, therefore, indicate the spread between the two specimens for each coating. The 10% variability between all the specimens was deemed acceptably low. It can be noted that there is greater spread in the thicknesses of the oil-infused coatings. This can be attributed to their lowered viscosity precuring, which can lead to leaking from the casting molds and greater variability in the final coating thickness. The results were corroborated by measurements with a micrometer.

#### Contact Angle

**Figure 8:**
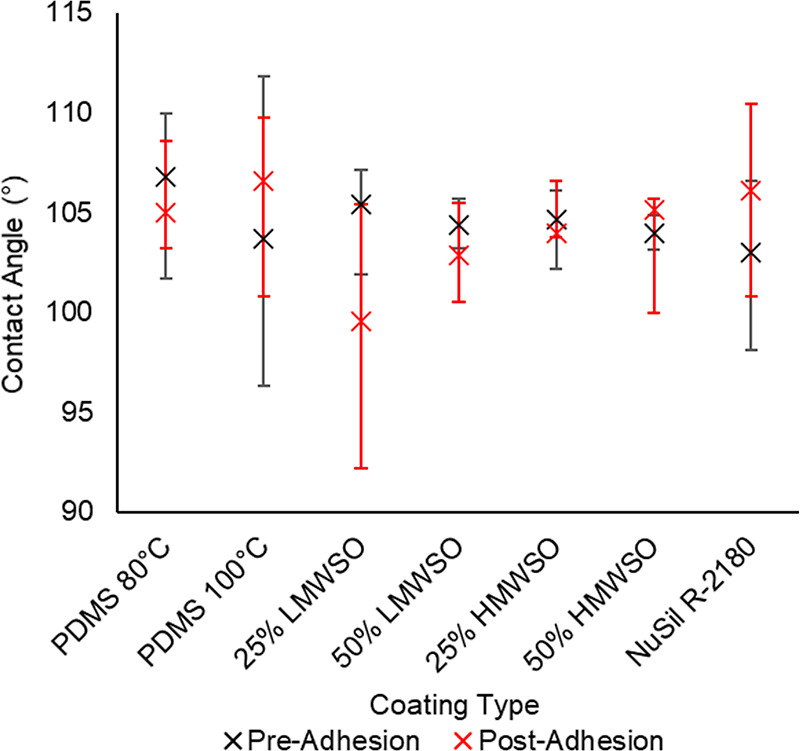
Contact angles on the coating types pre- and postadhesion testing, with range bars. Average values are calculated from two specimens per coating type, on which three droplets were placed and photographed. Each photograph was analyzed three times using the contact angle plug-in on ImageJ.

The results from the contact angle testing are presented in Figure 8. The average contact angle ranges from 100 to 107°. There is little difference between the coatings as the contact angle is dependent on the surface energy of the system, which is itself dependent on the chemical attraction between the water and the coating. The coatings have very similar chemistry, which is consistent with the very small differences in the observed contact angles.

The contact angles show no trends when comparing preadhesion results and postadhesion results: four coatings had greater contact angles postadhesion, and three coatings had smaller contact angles postadhesion. The range bars for both sets of data have considerable overlap, showing little significant difference between the pre- and postadhesion data. These results indicate that the effects of surface wear and dust accumulation that occurred during the adhesion regimen (see SEM images in Figures 11–13) were insufficient to meaningfully change the contact angle.

**Figure 9:**
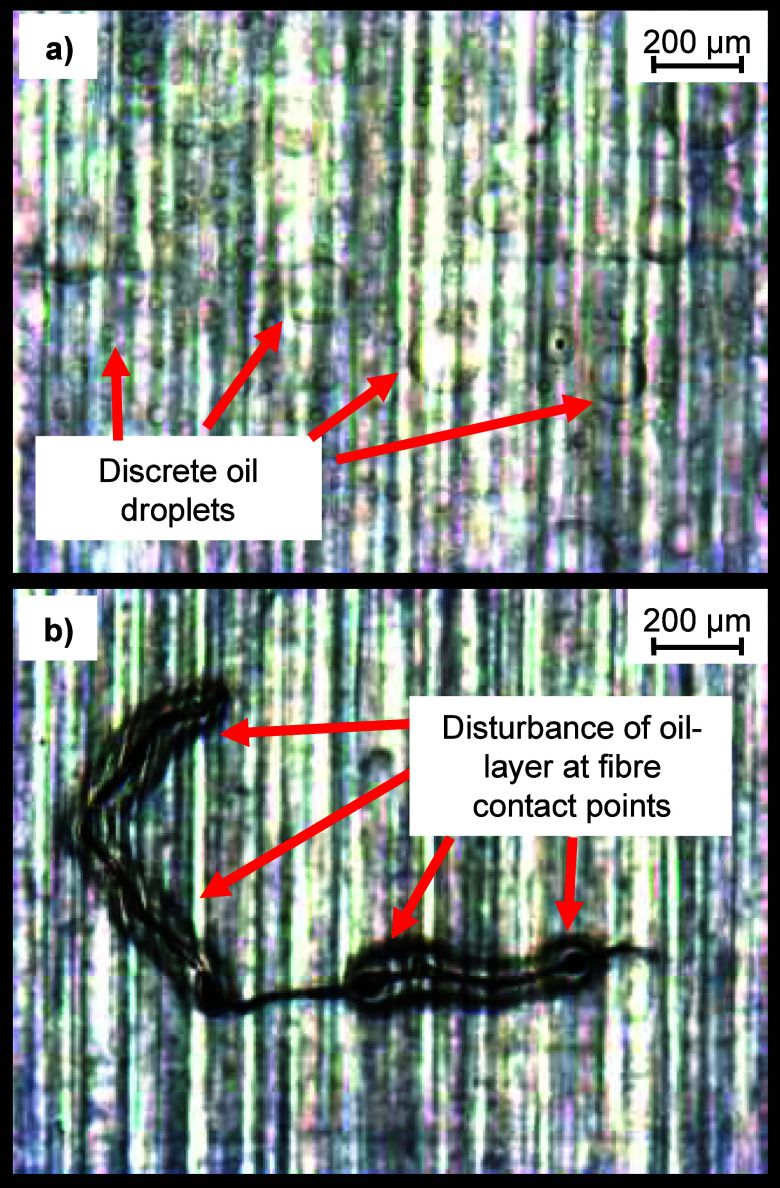
Optical microscopy images of surface oil on (a) 25% HMWSO and (b) 50% HMWSO. The 25% HMWSO surface has discrete surface oil, visible as droplets on the surface preadhesion. The 50% HMWSO surface has a continuous layer of oil, visible by the interaction of the dust fiber and oil, creating a halo effect at contact points.

**Figure 10:**
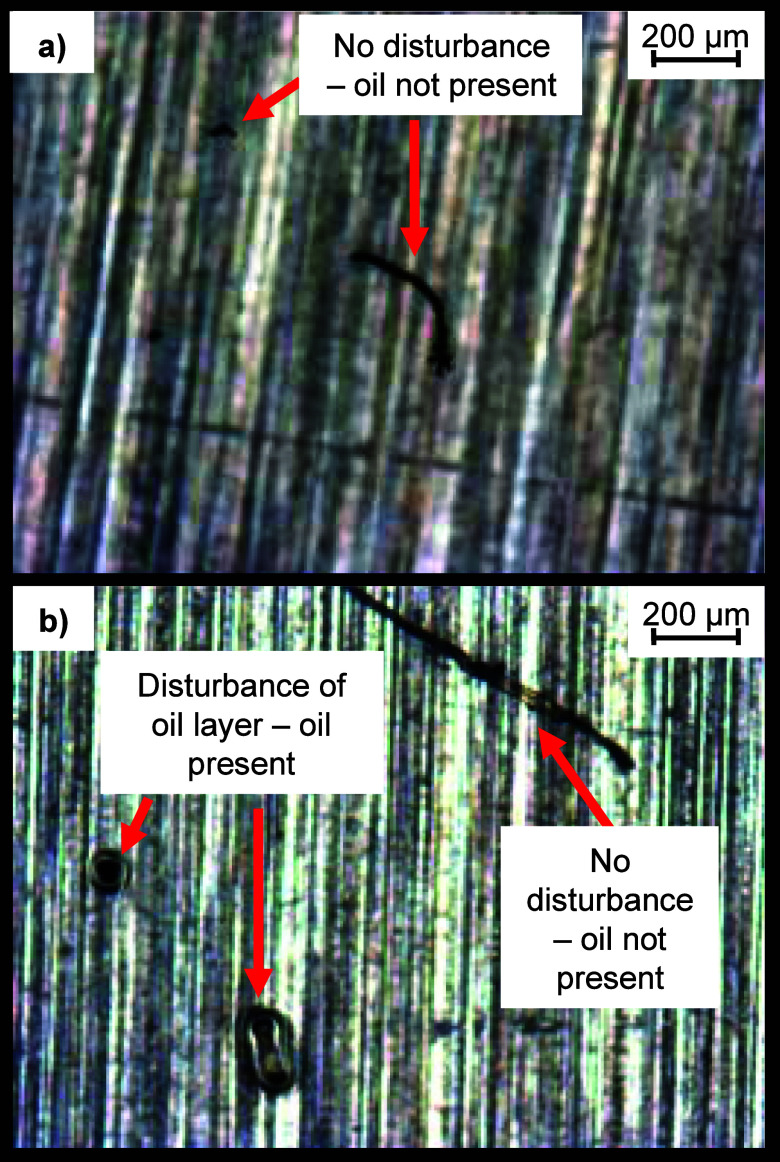
Optical microscopy images of surface oil on (a) 25% HMWSO and (b) 50% HMWSO postadhesion. 25% HMWSO specimen has no surface oil visible within the ice cylinder-contact area. 50% HMWSO specimen has some areas where an oil layer remains, shown by disturbance of the oil layer at dust contact points, but there are also some fibers/particles which do not have a halo around the contact points.

**Figure 11:**
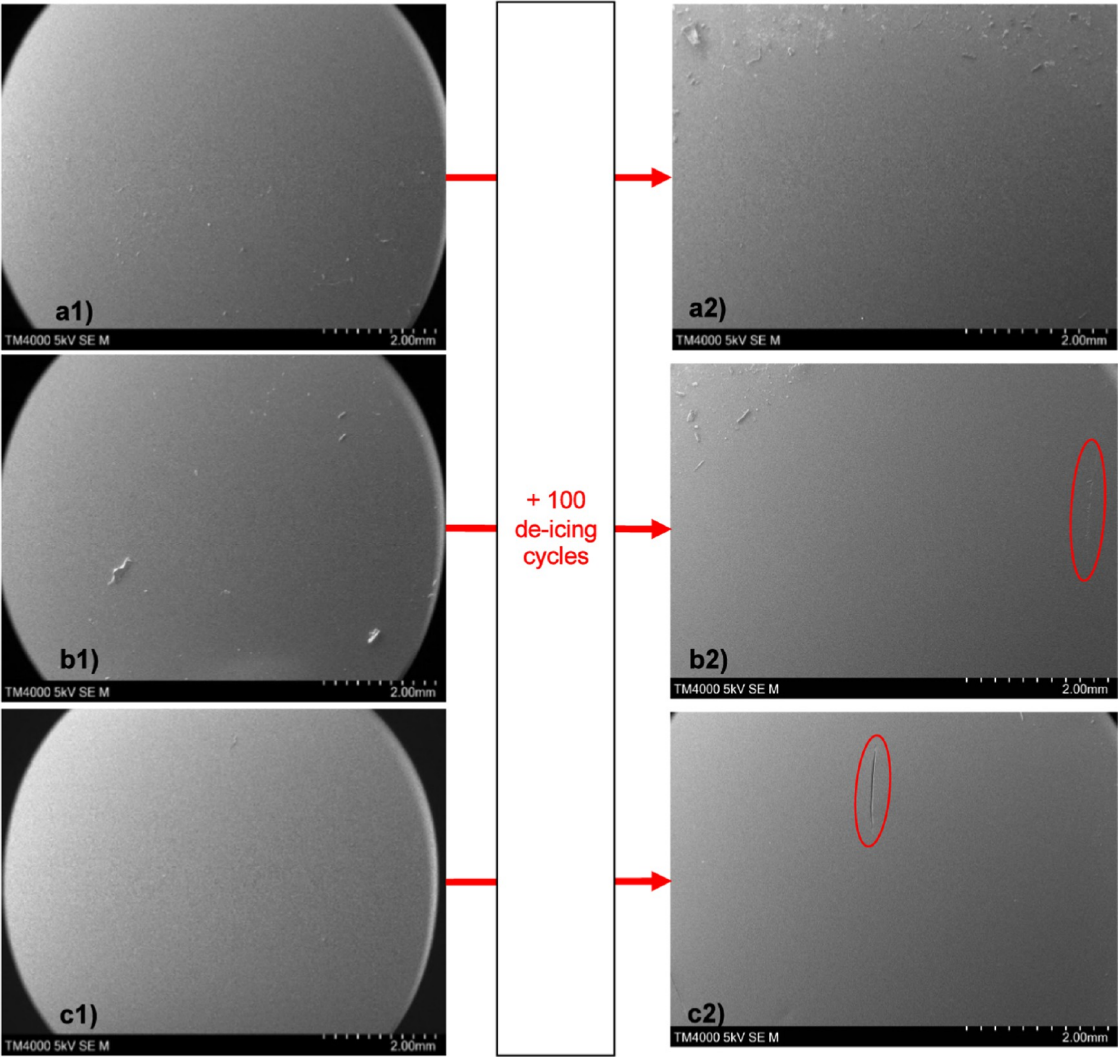
SEM images of coating surfaces pre- and postadhesion. The coatings are (a) PDMS 100 °C, (b) 25% LMWSO, and (c) 50% HMWSO. The only visible features on surfaces preadhesion are dust and fibers. Postadhesion surfaces show minimal damage, circled in red on (b2) and (c2) and shown in greater detail in Figure 13 (a,f), respectively. The circular contact area can be seen surrounded by dust at the top of (a2,b2).

**Figure 12:**
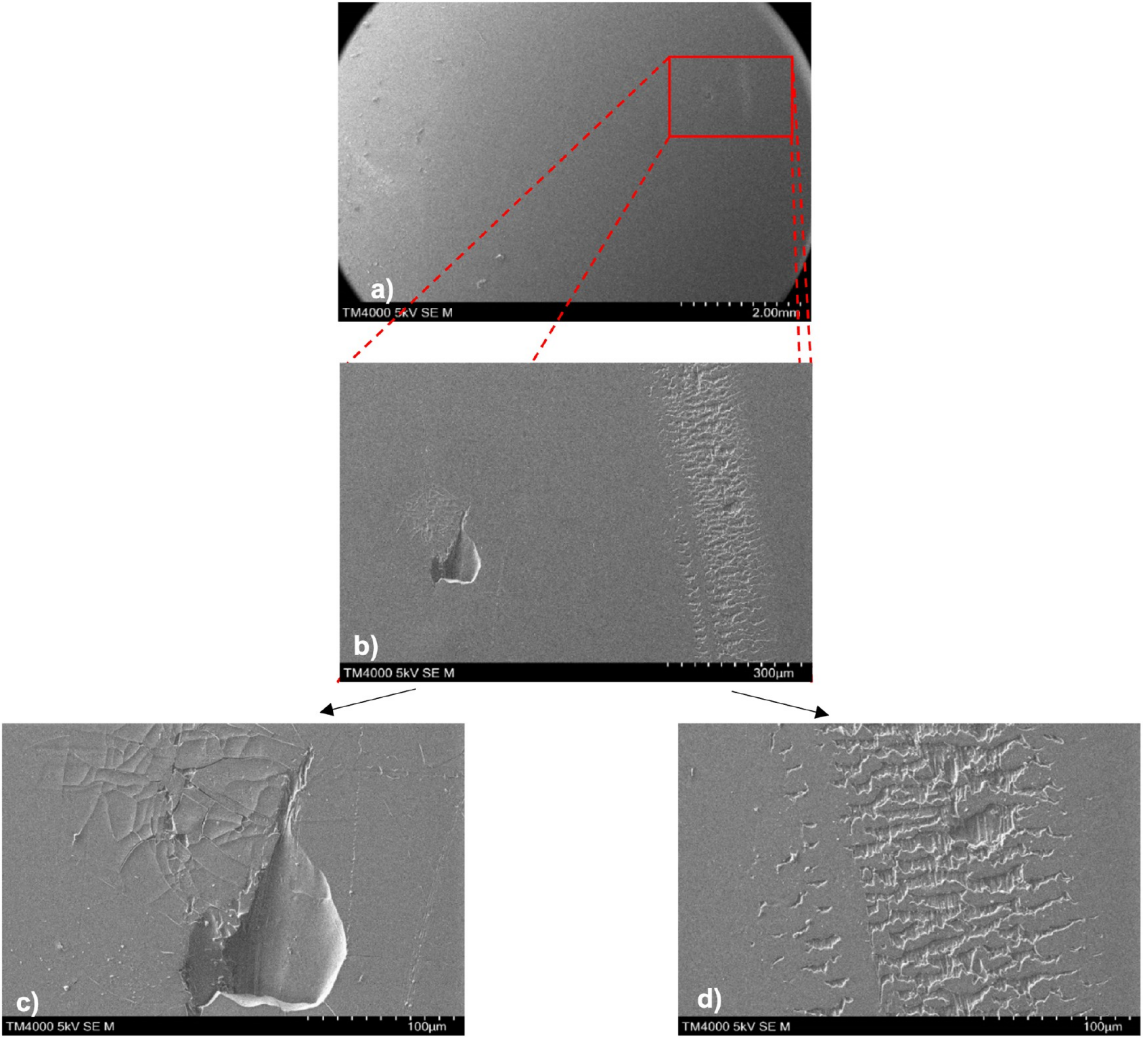
SEM images of a PDMS 80 °C specimen postadhesion. Panel (a) presents a representative view over a wide area with significant dust buildup on the left side of the image, and almost no dust in the center of the image, where the ice cylinder was placed for each adhesion test. Panels (b), (c), and (d) show higher magnification images, where damage has occurred on the surfaces. Panel (c) shows gouging damage that was common across the surfaces. It also shows cracking at the tip of the divot. Panel (d) shows abrasive wear damage.

**Figure 13:**
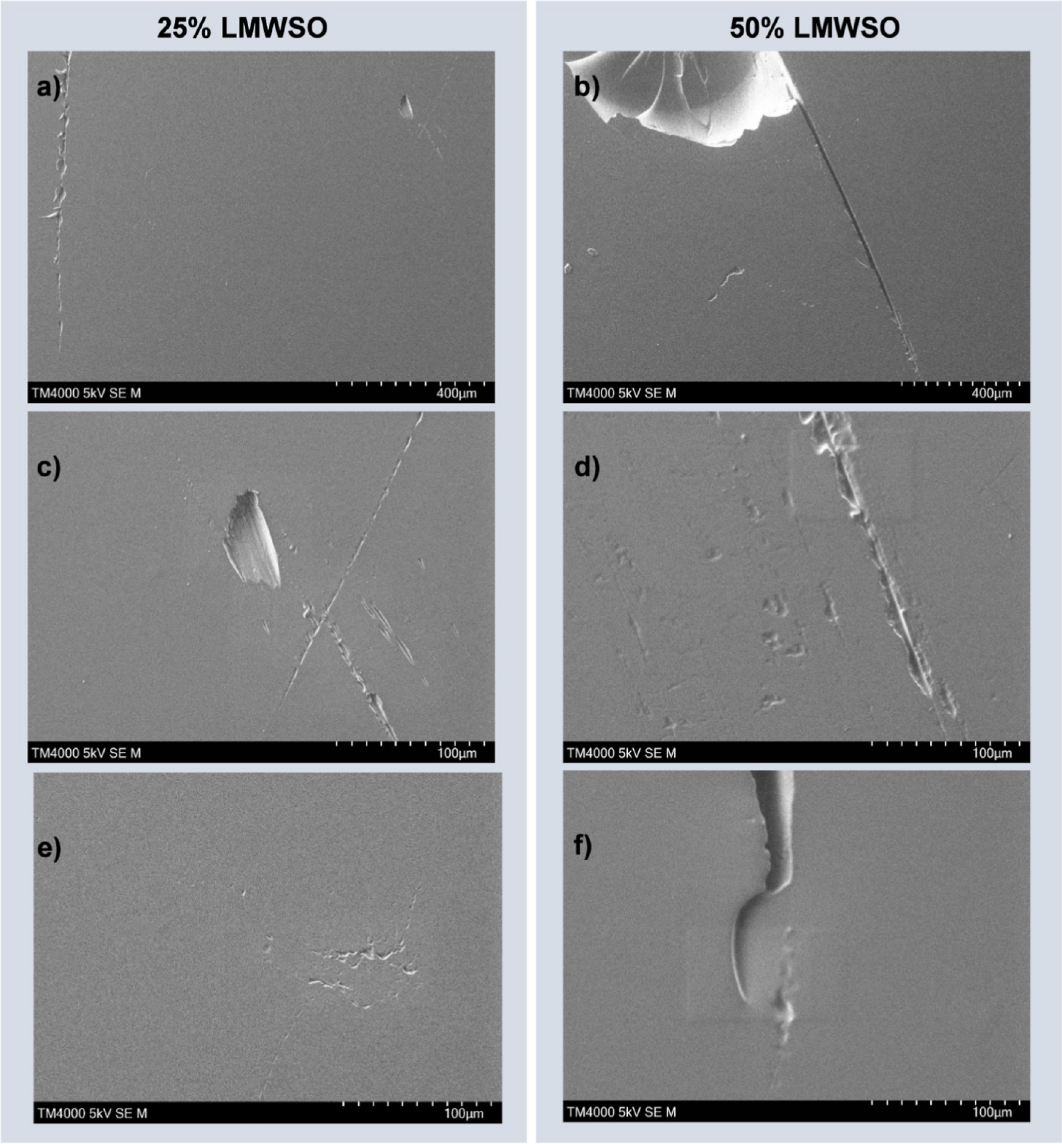
Examples of damage to LMWSO coatings after adhesion testing at different magnifications. Images show a mixture of cohesive gouging (a–c) and abrasive wear (a,d,e). Cracks in the surface (b,f) are stable and not forming networks. Incidental damage from impact by a screwdriver tip on one of the 50% LMWSO specimens is visible in panel (b).

#### Optical Microscopy

Optical microscopy was used to check for the presence of surface oil on the specimens. The only specimens which displayed surface oil were the 25% HMWSO and 50% HMWSO. Selected images of the specimens before adhesion testing are presented in Figure 9. On the 25% HMWSO specimens, there are clearly discrete droplets of oil across the surface. The presence of oil on the surface of the 50% HMWSO coating is less obvious. It is a continuous layer on the surface and is identified by the interaction of dust and dirt on the surface of the oil. Dust and dirt on the surface disturb the oil layer, and this is optically observable via the distortion of light around the point of contact. This appears almost as a halo around the particles/fibers, where they are in contact with the oil.

The specimens were also imaged after the adhesion testing (Figure 10). The images presented are within the ice cylinder contact area. The 25% HMWSO specimen has no surface oil visible, as either droplets or a continuous layer, within the contact area. However, outside of the contact area, there were still droplets of oil, which had not been removed from the surface by the ice detachment process. The 50% HMWSO specimen showed some evidence of an oil layer via the appearance of a halo around the dust particle contact points. However, the layer does not cover the whole surface—there are some fibers/particles which do not have a halo around them.

This evidence indicates there is oil on both surfaces before ice adhesion, with more oil on the 50% HMWSO, and that deicing removed the oil from the surfaces. The 25% HMWSO did not replenish the surface oil after testing had finished, while the 50% HMWSO was at least able to partially replenish the surface oil, likely due to the higher percentage of oil trapped in the bulk, which was able to migrate to the surface.

Repeating the optical microscopy at −10 °C gave similar results (see Supporting Information). In addition to the microscopy, the syneresis of oil onto the surface at −10 °C was investigated using a Kim wipe to blot the surface. Tests were performed in the cold laboratory at the following intervals after specimen fabrication: immediately, 1 day, 2 days, 8 days, 14 days, 30 days, and 135 days. Tests were performed after increasing time intervals, as it has been noted by Yeong et al.^35^ that oil migration to the surface can be on the scale of weeks. New icephobicity specimens were made for this testing, and the Kim wipes were placed over the entire surface and firmly pressed down to absorb any oil. Visual examination of the wipes was performed to observe any optical changes. Where oil was absorbed, the Kim wipe darkened and became translucent. The results are presented in Table S1 in the Supporting Information alongside sample images of Kim wipes, with and without absorbed oil (Figure S1). The results of this test agree with the findings of the optical microscopy: oil was primarily observed on the 25% and 50% HWMSO specimens, with fewer instances of oil on the LMWSO samples. Syneresis of oil onto the surfaces does not contribute significantly to the icephobicity of the LMWSO specimens at −10 °C. However, oil is present on the HMWSO specimens, and there is repeated replenishment of the oil from the bulk via syneresis.

#### Scanning Electron Microscopy

A selection of SEM images is presented in Figure 11. These images are representative of the findings on the coatings without surface oil (PDMS 80 °C, PDMS 100 °C, 25% LMWSO, 50% LMWSO, and NuSil R-2180). All the surfaces have minimal texture preadhesion, with no obvious roughness. The only features of note are dust and dirt on the surface. Images of the specimens after adhesion testing show little damage to the surfaces. Some instances of damage were identified and are presented in Figures 12 and 13.

The dust present on the specimens preadhesion is generally spread uniformly across the surface. After adhesion testing, the dust lies primarily around the edges of the images in Figures 11 and 12. The dust that settles within the bounds of the ice cylinder-contact area between tests is removed by detachment of the ice; ice detachment arguably ‘cleans’ the surface. In Figure 11, significant dust build-up occurred around the outside of the ice cylinder-contact area over the 100 tests.

Figure 12 shows postadhesion SEM imaging of a PDMS 80 °C specimen. The preadhesion surface was consistent with the other coating types. The postadhesion images highlight the two types of damage that were identified. There is characteristic elastomer abrasive wear^36,37^ in panel (d), as well as evidence for cohesive damage that caused gouging, or pitting, in the surface in panel (c). There is a gouge in the surface with a teardrop shape, as well as single tracks made from abrasion by debris at the left of the image. Crack networks have formed at the tip of the gouge in the surface. These cracks were only present in the pure PDMS surfaces, indicating reduced brittleness by the addition of oil. Pitting/gouging damage was the most seen damage type across all the specimens.

Further examples of damage to the LMWSO specimens are presented in Figure 13. These images show higher magnification views of both cohesive gouging (a, b, c) and abrasive wear (a, d, e). Though there is large crack formation in the surface (b, f), the cracks are stable and not forming networks. This may suggest that, despite being more susceptible to damage because of their softness, catastrophic brittle failure is better prevented by the addition of oil to the coatings.

Panel (b) in Figure 13 shows a 50% LMWSO specimen, which experienced incidental damage during adhesion testing when impacted by a screwdriver tip. This caused relatively large-scale damage to the surface when compared to the other damage events. The gouges created by this impact are significantly larger and deeper than the gouges created by ice detachment. It is likely that the damage affected the results of the icephobicity testing on this specimen, at the very least by changing the contact of the ice on the surface.

The imaging demonstrates that the ice detachment process generates minimal damage to the surfaces. This contradicts the common belief that the softness of the surfaces will lead to fast degradation from the stresses imposed during the detachment process.

### Ice Adhesion

**Figure 14:**
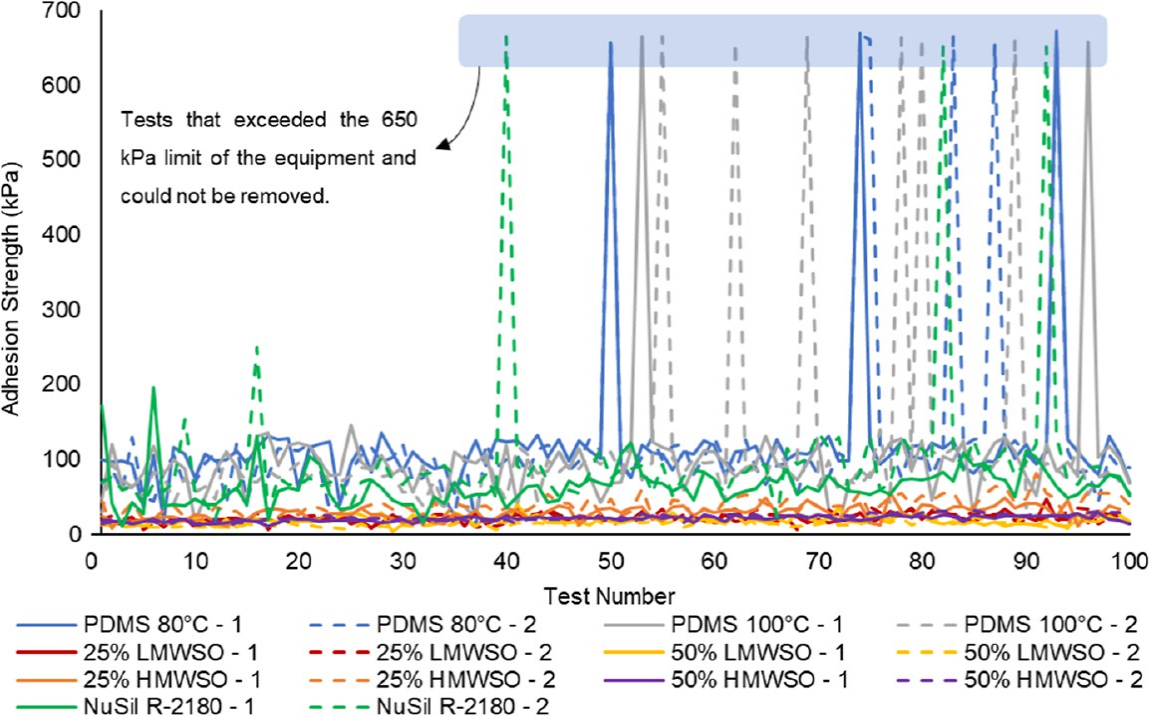
Ice adhesion strengths for each specimen over the testing regimen of 100 repeat deicing cycles. High outliers, in which the force required for detachment exceeded the load limit of the equipment (650 kPa), are highlighted at the top of the figure.

**Figure 15:**
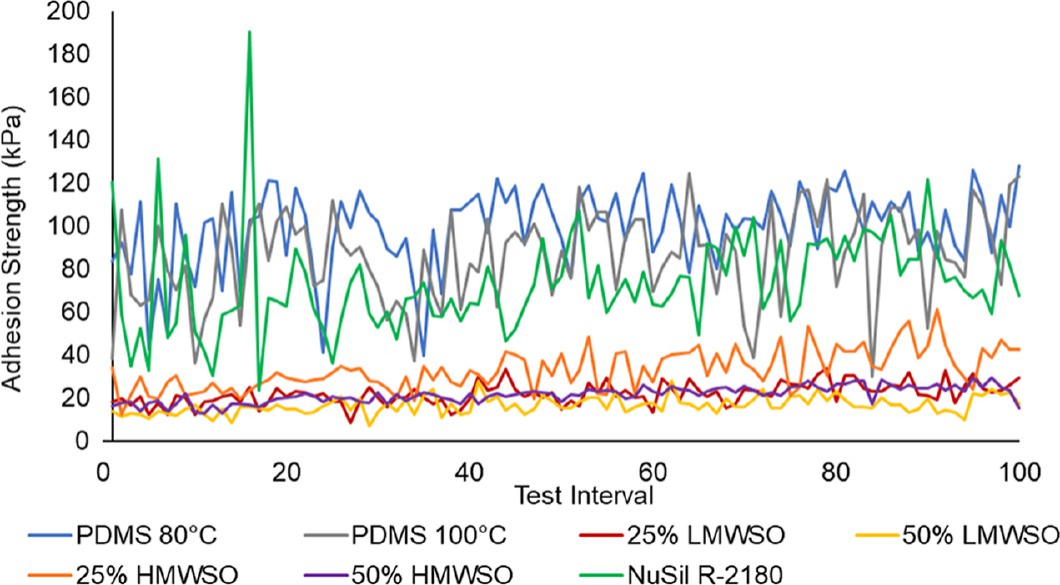
Ice adhesion strength of the different coatings over 100 repeat tests, carried out over approximately 9 months. The data is the average value of the two specimens for each coating. High outlier data has been replaced by additional tests.

Ice adhesion testing showed that, for 100 tests on each coating specimen, most of the measurements were below 200 kPa, except for a few tests which appear at 650 kPa. As shown in Figure 14, these high outlier tests in fact exceeded the 650 kPa load limit of the ForceBoard, and the ice could not be detached. To allow for consistent analysis of the coatings, additional testing was carried out to replace these tests. Using replacement tests, the average ice adhesion of both specimens for each coating across the testing period is presented in Figure 15.

Assessing the average values, adhesion strengths of the oil-infused coatings were approximately 5 times smaller than the noninfused coatings. The oil-infused coatings ≈20 kPa (the revised limit for icephobicity)^9−11^ and nonoil-infused coatings ≈100 kPa (the higher limit for icephobicity).^8^ This holds true for the duration of the testing. The NuSil R-2180 coatings showed a slight improvement on the PDMS coatings—73.2 kPa vs 99.3 kPa (80 °C) and 86.0 kPa (100 °C)—when taking the average of all tests. The 50% LMWSO performed best over the testing regimen, averaging 16.5 kPa. The 50% LMWSO coating even maintained an ice adhesion strength of less than 20 kPa in 79% of the tests and averaged under 20 kPa in the final 10 tests.

The addition of silicone oil reduces the ice adhesion strength significantly, and the greater the percentage of oil in the coating, the lower the ice adhesion. The addition of LMWSO reduced the adhesion more than HMWSO.

**Figure 16:**
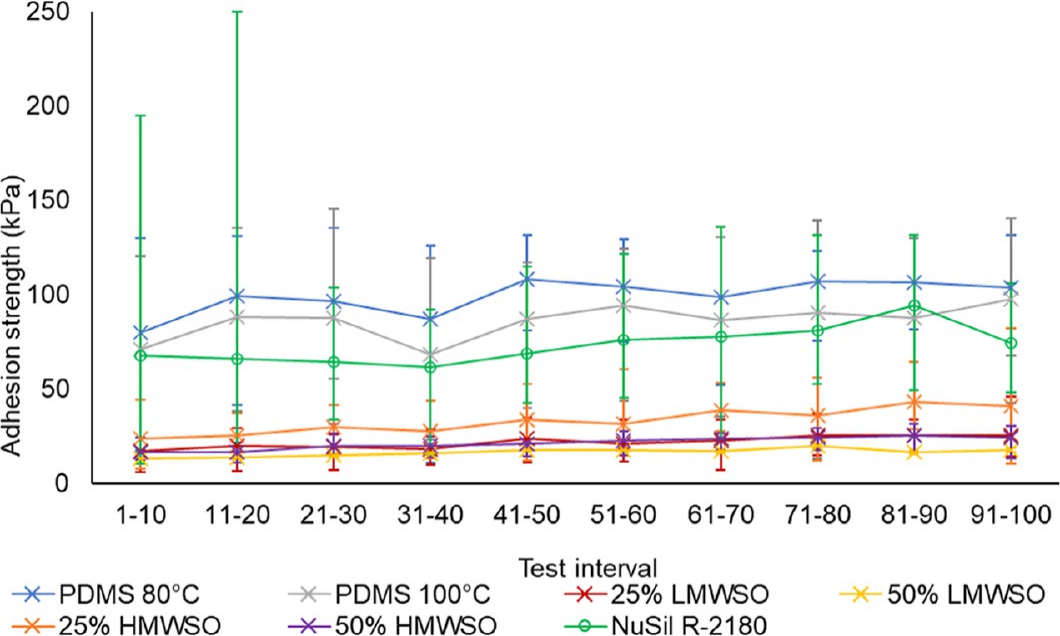
10-test average of ice adhesion strength for each coating type, with range bars.

**Figure 17:**
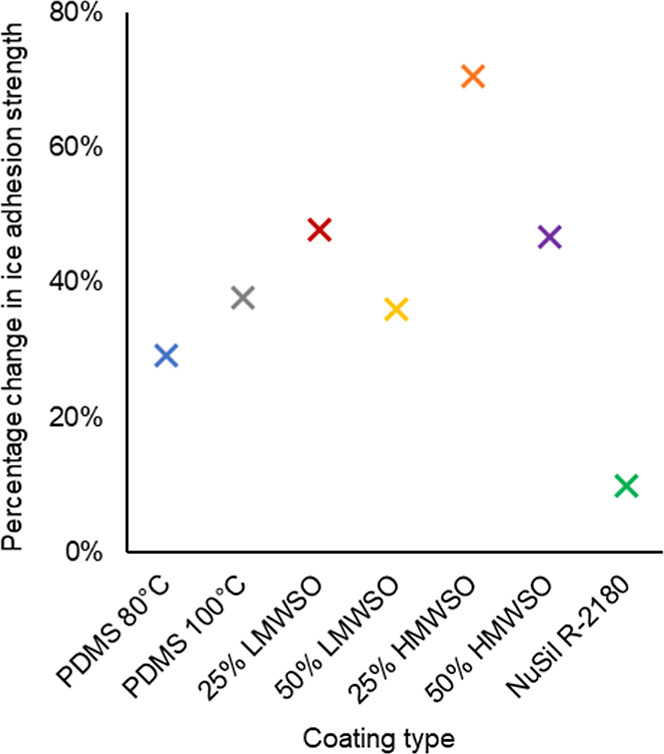
Percentage change in the 10-test average of ice adhesion strength between the first and last 10 tests for each coating.

Analysis of the 10-test average of each coating in Figure 16 shows the broader changes over the testing regimen, with a general increase in ice adhesion strength for all the coatings over the 100 tests. Assessing the final interval, the worst performing coating was the PDMS 100 °C with 103.6 kPa, and the best was the 50% LMWSO with 17.8 kPa. There was a minimum 10% increase in average adhesion strength from start to end (Figure 17). NuSil R-2180 had the lowest increase of 10%, and 25% HMWSO had the greatest increase of 71%. These results exclude the high outlier data points.

The data in Figures 16 and 17 show the softer, oil-infused coatings experience a greater percentage increase in adhesion strength, suggesting the softer a coating is, the faster it will degrade. Despite this, the oil-infused coatings maintained lower adhesion strengths than the nonoil-infused coatings over the entire testing regimen. The rate of degradation should not be viewed in isolation. Suitability to anti-icing applications should also consider the magnitude of the ice adhesion strength.

Furthermore, when combined with the SEM imaging in Figures 11–13, the ice adhesion results suggest the degradation or wear of the surfaces generated by the 100 deicing cycles is small and insufficient to cause large increases in the ice adhesion strength. Much of the concern with the application of elastomer coatings has been that they will degrade quickly.^10,17^ However, that is not the observation made here, and the coatings maintained low ice adhesion throughout the testing, particularly the oil-infused coatings.

#### High Outlier Data

**Figure 18:**
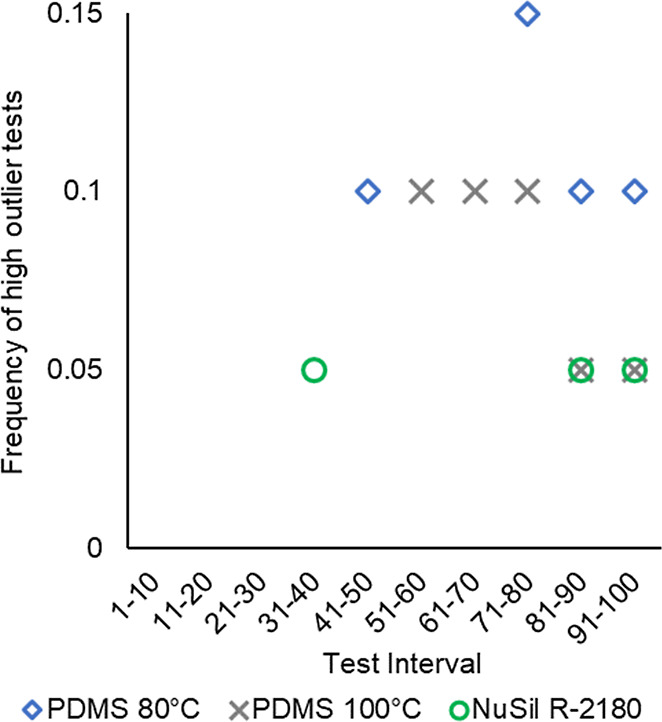
Rate of high outlier tests in nonoil-infused coatings (20 tests per interval). Notably, outliers never occurred in oil-infused coatings.

High outlier tests occurred for all but one of the nonoil-infused specimens and did not occur in any of the oil-infused specimens. In these tests, the specimen could not be removed by the ForceBoard, exceeding the test capacity of 650 kPa. The frequency of high outlier tests increased with repeat testing, as presented in Figure 18. The first occurrence was at test 40, but the rate rose to 7% for the noninfused coatings over tests 70–100. This trend suggests that performance is likely to continue worsening with further deicing cycles. The nonoil-infused coatings cannot be classified as icephobic at these instances, as they exceeded the defined maximum ice adhesion strength of 100 kPa. This behavior is not a singular outlier, but a tendency that increases over time. This is also not a gradual degradation, but a very sudden loss of anti-icing performance. Importantly, this limits the usability of these coatings in real applications, as the benefit they provide becomes inconsistent with repeated deicing.

Again, inspection of the wear surfaces in Figures 11–13 shows minimal damage, suggesting that wear is unlikely to be solely responsible for this drastic change in behavior. It is possible that a different mechanism governs the ice detachment in these tests. There may also be nanometer-scale modifications that affect the surface properties and adhesion, which we cannot exclude as they are not easily detected by SEM. The source of these high outlier tests remains an open question.

Generally, the higher the average adhesion strength (when excluding outliers), the more frequent the outlier behavior occurs. The oil-infused coatings also degraded, but degradation was gradual and never exceeded the load limits of the equipment. These findings reinforce the proposition that oil-infused coatings are a better choice for anti-icing applications than the nonoil-infused coatings, which can lose their icephobicity.

### Freezing Time

**Figure 19:**
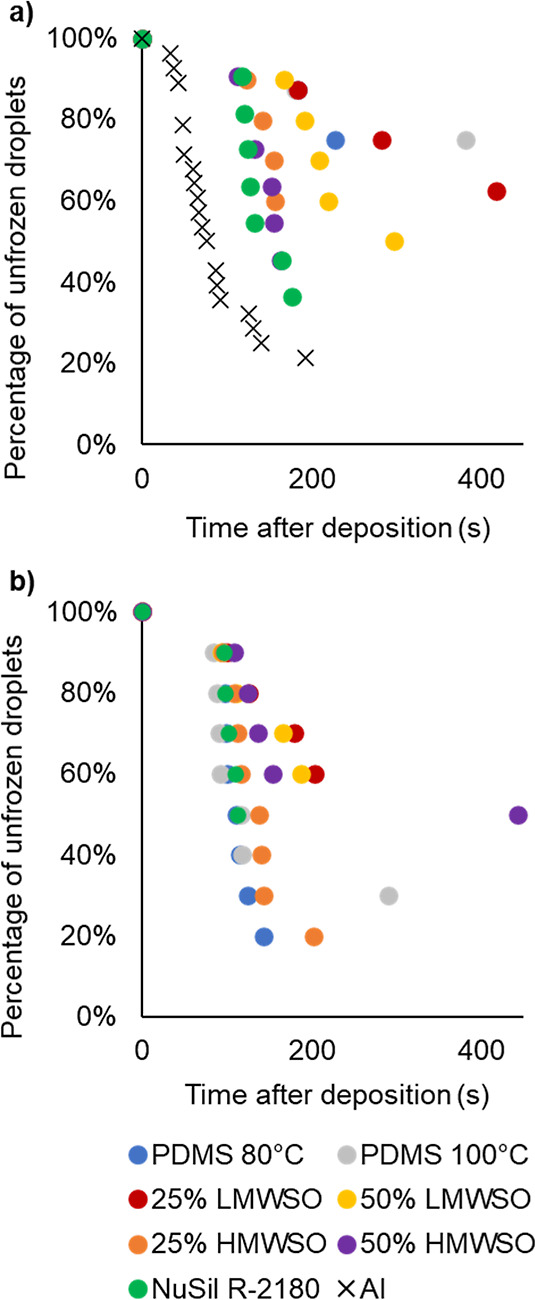
Freezing time statistics for water droplets on the coated surfaces (a) preadhesion and (b) postadhesion.

The measured freezing time for each test is presented in Figure 19. The graphs show the percentage of droplets on each surface which have frozen with progressing time. This statistical approach to reporting the freezing times has been utilized for droplet freezing studies at different temperatures,^38^ with aqueous alkali metal chloride solutions^39^ and charged diamond nanoparticles.^40^

The surface with the worst freezing times will have the steepest gradient in Figure 19, as droplets readily nucleate and freeze. As the gradient of the points becomes flatter, the surfaces have longer freezing times and are better at suppressing freezing. All the coatings exhibited improved freezing time compared to aluminum, which had a steep gradient and showed droplets readily nucleated on the surface. The coatings with the best freezing time (flattest gradients) preadhesion are PDMS 80 °C, PDMS 100 °C, and 25% LMWSO (Figure 19a). On each of these surfaces, only a few droplets nucleated during the test period. The NuSil R-2180, followed by both HMWSO coatings, demonstrated the least improvement to freezing time compared to aluminum. The 50% LMWSO had moderate improvement. Additional analysis of the average freezing times and the frequency of supercooling is provided in the Supporting Information.

The postadhesion data (Figure 19b) shows a broad decrease in the surfaces’ freezing time, and the curves have a steeper decline. The detachment process therefore alters the surface to increase the nucleation frequency. The coatings which show the best freezing time postadhesion are the 25% LMWSO and 50% LWMSO. Though the PDMS 80 °C and PDMS 100 °C had excellent freezing time preadhesion, they showed the weakest performance postadhesion, making them less durable with respect to delaying nucleation. The Nusil R-2180 and HMWSO coatings show weak improvement to freezing time pre- and postadhesion testing. Considering the pre- and postadhesion data together, the coatings with the best freezing time are the 25% LMWSO and 50% LMWSO, as they show the best improvement and are durable, maintaining improvement even after 100 detachment cycles.

The improvements to freezing time on the coatings compared to aluminum can likely be attributed to the statistical behavior of nucleation and the different thermal properties of the metal compared to the polymers. The droplet-coating systems in this study undergo heterogeneous nucleation, i.e., the ice nuclei form at the substrate–water interface rather than homogeneously within the water droplet. Ice nuclei form on nucleation sites on the substrate; these nucleation sites can be described as external nuclei. For a given temperature, the steady-state heterogeneous nucleation rate per unit surface area on external nuclei type i, is denoted by *J*_i_^st^ and has been shown to be largely dependent on the contact angle of ice on the surface within supercooled water, θ_is(w)_. As θ_is(w)_ approaches 180°, *J*_i_^st^ approaches a minimum equivalent to the homogeneous nucleation rate.^41^ This relationship has been derived via classical nucleation theory and is explained in full in the literature.^42^ The overall likelihood of nucleation occurring within a given time for a droplet on a surface is therefore dependent on θ_is(w)_ and the number of nucleation sites.

Once nucleated, the time taken for the droplet to completely freeze is dependent on the rate of heat transfer from the droplet. In this laboratory environment with little airflow around the droplet, heat transfer is controlled by the thermal conductivity and thickness of the substrate, through which most of the heat is lost.^7^

Compared to aluminum, the coating surfaces are likely to have greater values of θ_is(w)_ and fewer nucleation sites due to their hydrophobicity and relative smoothness. This would explain the lower nucleation frequency observed. The lower thermal conductivities of the elastomers^43,44^ and silicone oil^45,46^ and increased substrate thickness compared to aluminum will extend the growth phase of the ice and thus also the total freezing time.

The greater nucleation frequency across the coatings postadhesion testing is likely a result of the increasing number of nucleation sites, from surface damage caused by the deicing cycles or dust accumulation, rather than a change in the value of θ_is(w)._

Having very similar hydrophobicity (indicated by the static contact angles in Figure 8), the different coatings are likely to have very similar values of θ_is(w)_. The silicone oil and PDMS also have similar thermal conductivities, so they will have similar growth times. Therefore, large differences observed in freezing time between the coatings are likely due to different numbers of nucleation sites on the surfaces. This can be demonstrated in the observation that after adhesion testing, the differences in freezing time are much smaller, as the number of nucleation sites increases to saturation and the freezing time trends to the theoretical minimum. This is composed of the minimum time lag for ice nuclei formation in the system and the ice growth time. The minimum time lag is again dependent on θ_is(w)_. The postadhesion data shows the curves converging toward similar gradients—the minimum freezing time. As the minimum freezing time is expected and shown to be similar between the coatings, large differences in freezing times observed are likely attributable to different nucleation site densities. Differences in θ_is(w)_, the substrate thickness or thermal conductivity may be responsible for small differences.

As stated above, the best coatings when considering the freezing times and preservation of freezing time after deicing cycles are the 25% LMWSO and 50% LMWSO. Further investigation may elucidate the source of this, whether it is (i) some combination of small differences in θ_is(w)_, the substrate thickness or thermal conductivity, or (ii) a resistance to the formation of new nucleation sites. This could be probed by performing additional deicing cycles and freezing time tests until the minimum freezing times are identified and compared across the coatings. Regardless, all the coatings remain better than aluminum, so their application would reduce ice nucleation rates compared to uncoated aluminum, and oil infusion is shown not to be detrimental.

### Ice Detachment Models

The adhesion data were fitted to an interfacial cavitation^47,48^ model for the detachment of a rigid solid from an elastic substrate to predict the ice adhesion strength. Interfacial cavitation assumes separation occurs by the propagation of a wave of air cavities along the interface during detachment and is described by the relationship
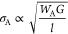
2where *W*_A_ is the work of adhesion between the two surfaces, *G* is the shear modulus, and *l* is the thickness of the coating. *W*_A_ is defined via the Young-Dupré equation^49^ as
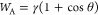
3where γ is the water–air surface tension and θ is the static water contact angle on the coating. For calculation purposes, the surface tension at 20 °C was used to remain consistent with the temperature at which the contact angle was measured. A value of 0.07275 N/m was used for γ.^50^ Shear modulus, *G*, was calculated from the elastic modulus and Poisson’s ratio, ν, via the relationship for a homogeneous, isotropic material
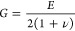
4

Values of Poisson’s ratio for PDMS are normally quoted from 0.48 to 0.5.^51−53^ For the purposes of this study, a Poisson’s ratio of 0.5 was assumed, as has been adopted in other studies.^54,55^ Additionally, the viscoelasticity of the polymers was investigated using dynamic mechanical analysis (DMA) as Poisson’s ratio can decrease significantly near the glass transition temperature.^55^ The details of this testing are provided in the Supporting Information, but Figure S6 demonstrates that, for all the polymers, the storage moduli are much greater than the loss moduli at both room temperature (20 °C) and the icephobicity test temperature (−10 °C). Therefore, the mechanical response at both temperatures is dominated by the elastic behavior, and a Poisson’s ratio of 0.5 is sufficient for this study. It should be noted that for plain PDMS, the glass transition temperature (*t*_g_) has been shown to be below −100 °C,^56−58^ with crystallite melting at approximately −50 °C.^57−59^

**Figure 20:**
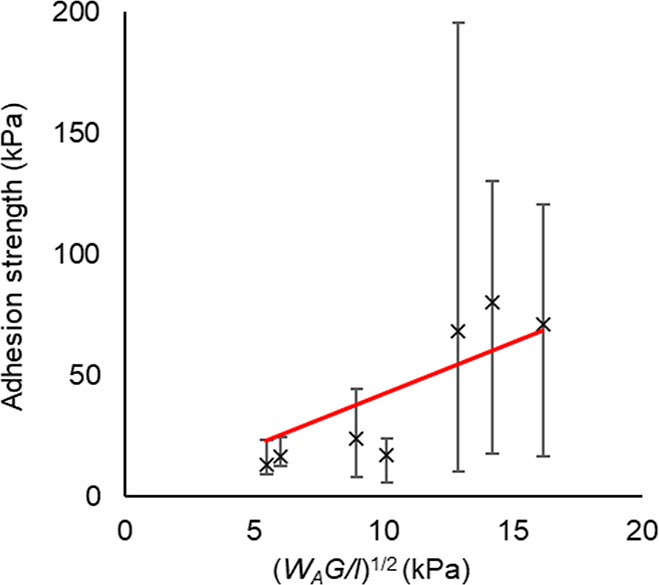
Dependence of ice adhesion on work of adhesion, shear modulus, and coating thickness, as proposed in eq 3. A linear fit provides a correlation coefficient of 0.70. The proportionality constant is 4.16.

Examination of the relationship described by eq 2 in Figure 20 shows a good linear fit. Fixing the intercept at (0,0) gives a correlation coefficient of 0.70. An initial assessment indicates a good possibility of interfacial cavitation.

**Figure 21:**
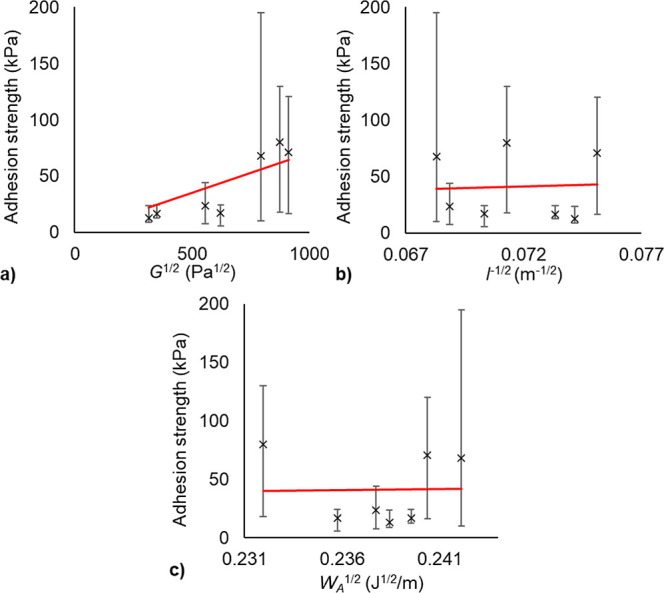
Evaluation of the relationship between ice adhesion and variables from the interfacial cavitation model (a) shear modulus, (b) coating thickness, and (c) work of adhesion. The relationship between ice adhesion strength and shear modulus is the strongest; there is negligible correlation between ice adhesion and coating thickness or work of adhesion for these coatings.

Further assessment of the variables of *W*_A_, *G*, and *l* in Figure 21 demonstrates the contribution of each to the ice adhesion behavior. For *l*^–1/2^ and *W*_A_^1/2^, it is observed there is negligible correlation between either of these terms and ice adhesion strength. In contrast, ice adhesion and *G*^1/2^ also have a correlation coefficient of 0.70. Of the properties considered here, the ice adhesion strength of the coatings is most influenced by shear modulus. This is explained by considering the extent of variability in shear modulus compared to thickness or work of adhesion. The largest value for shear modulus is nearly 10 times greater than the smallest, while the variation in thickness and work of adhesion is minimal.

The practicality of altering either thickness or work of adhesion is limited. The work of adhesion, as a function of contact angle, is dependent on the chemical composition of the coating, which affects the physicochemical interactions of the surface. In this system, the bonding is predominantly physical, with minimal chemical bonding. Extensive changes to the chemistry of the coatings will also affect physical properties, like the shear modulus, as well as the processability, cost, and environmental impact. Making minor adjustments, like changing the polymer chain length in this work, has a limited effect on the work of adhesion.^33^

The ability to affect the ice adhesion strength by controlling the thickness of the coating is limited by the range of thickness for which the relationship holds true. It has been shown that there is a critical thickness, above which the ice adhesion is independent of thickness. Literature suggests this is in the order of 0.5 mm^10,60,61^ and therefore the lowest ice adhesion strengths achievable for a given coating occur around this thickness. However, increasing coating thickness will also increase the weight and cost of a system. The benefits in reducing ice adhesion strength must be balanced against the added weight, which is of great importance in some systems.

**Figure 22:**
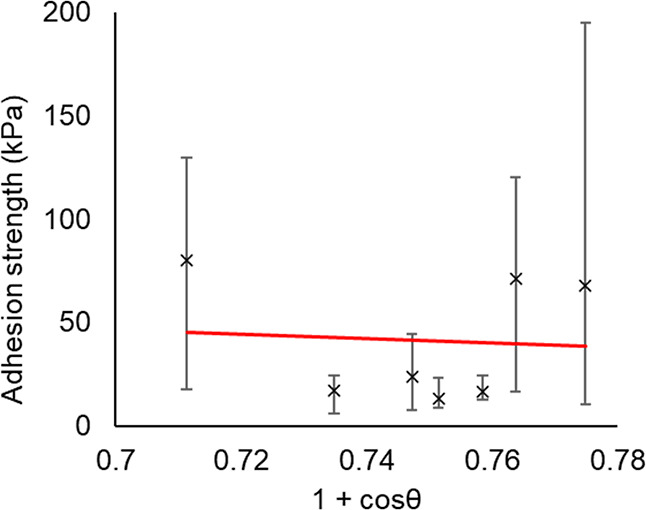
Relationship between ice adhesion and 1 + cosθ. The results show no correlation.

As already shown, *W*_A_^1/2^ does not show strong correlation with the ice adhesion strength. Additional analysis has been performed in the literature showing moderate correlation between ice adhesion strength and *W*_A_ directly. Meuler et al. reported a correlation coefficient of 0.8 for ice adhesion and (1 + cosθ).^62^ Performing the same analysis in Figure 22 shows no correlation for these coatings. Meuler et al. examined coatings with a much greater variation in chemistry and contact angle, but which encompassed the values of (1 + cosθ) measured in this study.

It can be concluded that static contact angle measurements are not a useful indicator for ice adhesion among PDMS-based elastomers. It should be noted that some studies have found better correlation between ice adhesion and dynamic water contact angles.^61−64^ However, there is still a lack of consensus on which term, if any,^65^ best describes ice adhesion strength. The roll-off of a liquid (water) from a solid surface poorly reflects the detachment of a solid (ice) due to the fundamental mechanical differences of the interfaces,^8,25^ so we argue liquid contact angles should not be used as a primary predictor for ice adhesion strength. The static contact angles are provided to demonstrate that the underlying hydrophobicity and surface energy of the specimens are very similar, and these factors only contribute a small part to the observed ice adhesion differences of our surfaces.

For these coatings, the dependence on shear modulus is the most significant. Of the terms included in this model, ice adhesion strength can be best predicted via the shear modulus, specifically the square root of the shear modulus. From a physical perspective of the findings: the lower the shear modulus of the coating, the bigger the mismatch between the ice’s modulus and the coating’s modulus. This leads to larger strains in the coating when force is applied at the interface, which cannot be matched by the ice, and hence, the ice more easily detaches from coatings which have lower shear moduli—as we see in the results.

## Conclusions

Silicone elastomers were fabricated for anti-icing coatings, using PDMS and NuSil R-2180. PDMS prepolymer mixtures were combined with silicone oil of two molecular weights and percentages to produce oil-infused coatings. The icephobicity of the coatings was measured via the ice adhesion strength and freezing time, with room-temperature measurement of contact angle, coating thickness, elastic modulus, and imaging performed via optical and scanning electron microscopy.

The oil-infused coatings had significantly lower ice adhesion strengths compared to the nonoil-infused coatings—regularly less than 50% of the nonoil-infused coatings. Additionally, the nonoil-infused coatings had increasing instances in which the 650 kPa load limit of the equipment was exceeded, making them not reliably icephobic in the long term. This increase was likely not attributable to surface wear exclusively, which was shown in the SEM imaging to be minimal. Though surface damage was slightly greater on the softer, oil-infused coatings, it was not detrimental to the ice adhesion strengths over the testing period, which remained below 100 kPa. The 50% LMWSO coating performed best, with an average adhesion strength of 16.5 kPa over 100 tests.

As well as providing significantly better ice adhesion, oil infusion did not negatively affect the freezing time of the coatings, which provide an improvement on uncoated aluminum even with surface wear from 100 deicing cycles. 25% LMWSO and 50% LMWSO had the best combination of long freezing times and durability.

Analysis of the ice adhesion data via a cavitation detachment model showed adhesion strength is much more strongly influenced by shear modulus, *G*, than coating thickness, work of adhesion, or static water contact angle. The modulus is negatively correlated to the oil percentage but is not strongly influenced by oil molecular weight.

The application of oil-infused silicone elastomer coatings would reduce ice accumulation on surfaces compared to nonoil-infused coatings and bare metals, lowering the energy, time, and resources spent on deicing and the associated risks. Wear of the coating from deicing is minimal and does not significantly affect icephobicity. These coatings are promising for practical icephobicity applications.

## Supporting Information Available

Syneresis testing of oil-infused coatings; additional freezing time analysis; and DMA of polymers (PDF)

## Abbreviations

DMA: dynamic mechanical analysis
DSLR: digital single-lens reflex
HMWSO: high molecular weight silicone oil
LMWSO: low molecular weight silicone oil
PDMS: polydimethylsiloxane
PTFE: polytetrafluoroethylene
SEM: scanning electron microscopy
SHS: superhydrophobic surfaces
SLIPS: slippery liquid-infused porous surfaces.
