# Supplementary material for: Icephobicity of Oil-Infused Silicone Elastomer Coatings: Ice Adhesion, Freezing Time, and Room-Temperature Characterization
Source: ACS Omega. 2025 Apr 8;10(15):15681–96. doi: 10.1021/acsomega.5c01139 (PMC12019500; doi:10.1021/acsomega.5c01139)
Supplement: Supplementary file 1 — ao5c01139_si_001.pdf [file ao5c01139_si_001.pdf]

Icephobicity of Oil-Infused Silicone Elastomer Coatings: Ice Adhesion, Freezing Time and Room-Temperature Characterization

Catherine M. Megregian[1], Vasileios Koutsos[1], Anthony Callanan[2], Jane R. Blackford[1]*

[1]School of Engineering, Institute for Materials and Processes, The University of Edinburgh, Sanderson Building, King's Buildings, Edinburgh EH9 3FB, U.K.

[2]School of Engineering, Institute for Bioengineering, The University of Edinburgh, Faraday Building, King's Buildings, Edinburgh, EH9 3DW, U.K.

*Email: jane.blackford@ed.ac.uk

SUPPORTING INFORMATION

SYNERESIS TESTING

METHOD

Oil-infused icephobicity samples were fabricated using the method described in the Experimental Section. Two samples of each formulation were made (25% LMWSO, 50% LMWSO, 25% HMWSO and 50% HMWSO). After fabrication the samples were placed in the cold laboratory (–10ºC ± 1ºC, 80% ± 10% RH). Immediately after placement into the cold laboratory, and at 1, 2, 8, 14, 30 and 135 days, the surfaces were carefully blotted with a Kim wipe to absorb any surface oil. The presence of oil was the indicated by a change in the

transparency of the wipe where the oil was absorbed. Examples of Kim wipes with and without absorbed oil are presented in Figure S1.

Additionally, optical microscopy was repeated on the surface before the final test at 135 days from fabrication – 105 days after the previous test. This testing was performed in the cold laboratory at −10°C.

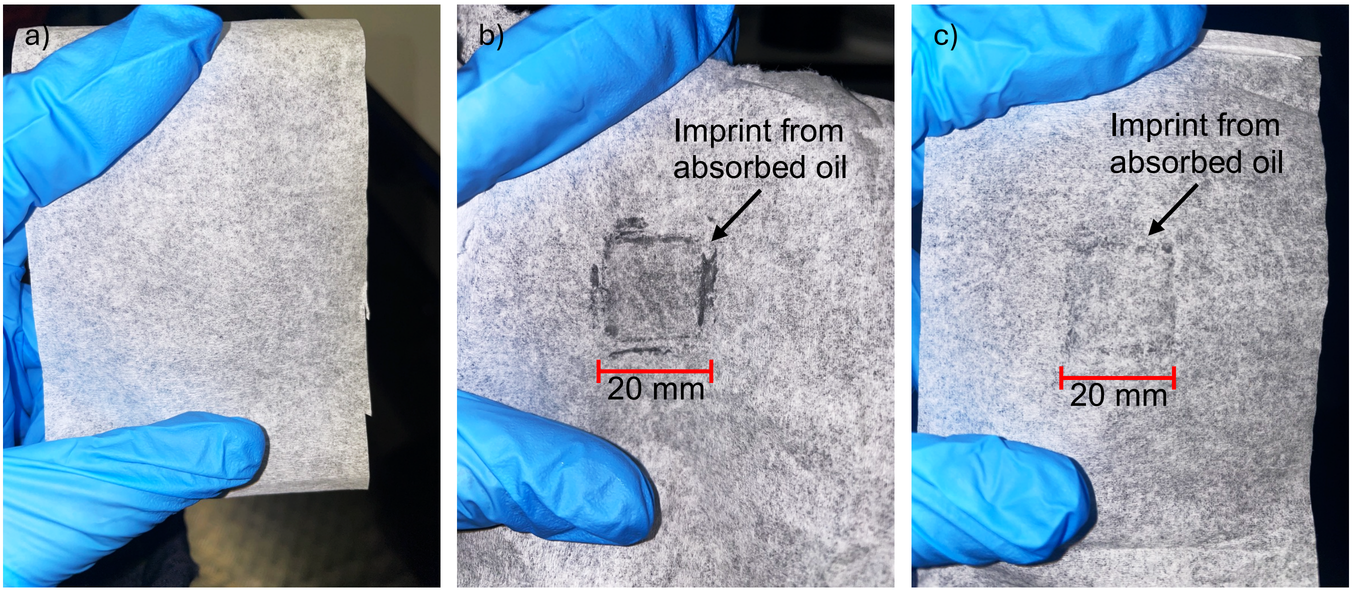

Figure S1. Photographs of Kim wipes during blotting tests. Panel a) shows the 25% LMWSO sample 1 after 2 days and has no oil absorbed onto the paper. Panel b) shows the 50% HMWSO sample 1 immediately after fabrication and c) shows the 50% HMWSO sample 2 after 14 days. Panels b) and c) demonstrate oil absorbed from the surface in the square shape of the coating specimen.

RESULTS

The results from the blotting tests are presented in Table S1. The LWMSO samples have minimal surface oil, particularly the 25% specimens, which only showed the presence of oil once per specimen. The 50% LMWSO specimens appear to have limited free oil reserves, as the blotting tests indicated there was largely no replenishment of oil from syneresis after being blotted three times. Furthermore, there was no syneresis between the blotting tests on day 30 and day 135; even after 105 days at −10°C oil was not expressed from the bulk. This means

that, after a few de-icing cycles, the observed icephobicity of these surfaces is not attributable to the presence of oil on the surface. Surfaces that utilise surface oil for low ice adhesion often exhibit drastic increases in ice adhesion when the oil reserves are depleted. This sudden increase in ice adhesion should not occur on the LMWSO surfaces, making their ice adhesion behaviour more reliable for anti-icing applications.

Table S1. Presence of oil on surface (x) as indicated by blotting tests at specified days after fabrication. Samples were left in the cold laboratory for test duration.

	**25% LMWSO**		**50% LMWSO**		**25% HMWSO**		**50% HMWSO**	
**Day**	**Sample 1**	**Sample 2**	**Sample 1**	**Sample 2**	**Sample 1**	**Sample 2**	**Sample 1**	**Sample 2**
**0**			x	x		x	x	x
**1**		x	x	x		x	x	x
**2**			x	x		x	x	
**8**						x	x	x
**14**	x				x		x	x
**30**				x	x	x	x	x
**135**					x	x	x	x

On the contrary, the HMWSO surfaces showed regular and continued syneresis. One of the 25% HMWSO specimens remained oil-free until 14 days after fabrication, suggesting that the syneresis rate may be slow, even at –10°C. However, each successive blotting test after 14 days showed the presence of oil and the other specimen showed oil on the surface in all but one test. Therefore, syneresis is likely to occur on 25% HMWSO coatings and surface oil will contribute to the icephobicity observations in this study. Though the replenishment limit has not been identified during these experiments, it cannot be limitless and the coatings may exhibit a large increase in ice adhesion strength during their service life, as noted above. The same can be said about the 50% HMWSO coatings, which had surface oil on almost every blotting test. The more regular instances of surface oil are likely because of the higher concentration of free oil in the bulk available to migrate to the surface and faster syneresis.

The results of the microscopy (Figure S2) agree with the observations of the blotting tests. There was some oil present on the 25% HMWSO surfaces, and a continuous oil layer on the 50% HMWSO even after 6 instances of blotting.

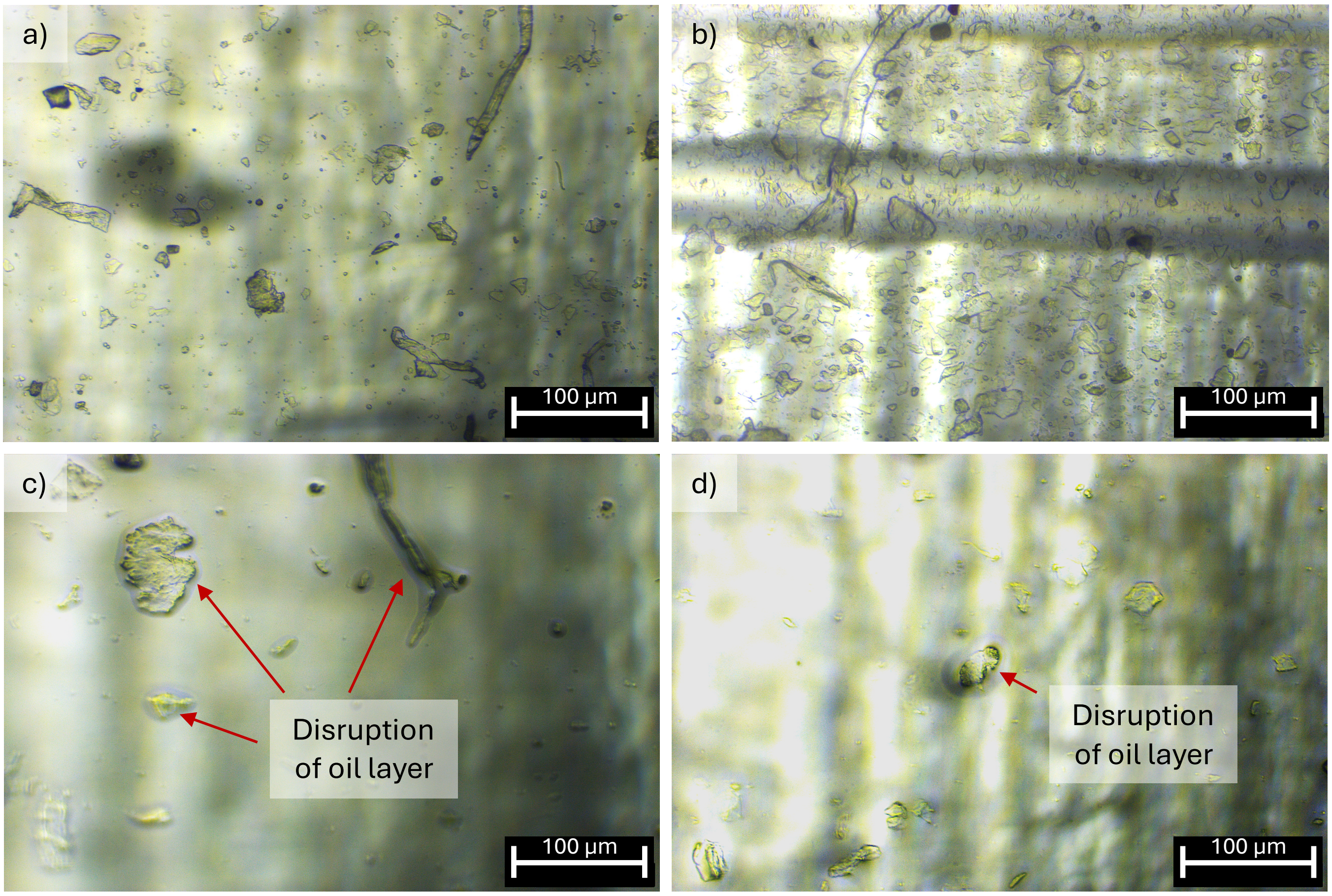

Figure S2. Optical micrographs of specimens before final blotting test. Panel a) and b) show no halo-effect around the surface debris on a 25% LMWSO and 50% LMWSO specimen, respectively, indicating no oil is present. Panels c) and d) show disruption of an oil layer via the halo-effect around the surface debris on a 25% HMWSO and 50% HMWSO specimen, respectively.

Our findings indicate there are sufficient oil reserves to replenish the surface oil after 100 de-icing tests or seven blotting tests over 135 days. Due to the possibility of a sharp increase in ice adhesion strength when the oil is depleted, the HMWSO coatings may be less reliable long-term anti-icing solutions.

ADDITIONAL FREEZING TIME ANALYSIS

The average freezing time for the surfaces was calculated and plotted in Figure S3. The coatings all provided an improvement in average freezing time compared to aluminum, with a minimum 51 s increase (59%) pre-adhesion and a 17 s increase (20%) post-adhesion testing (both NuSil R-2180), as shown in Figure S3.

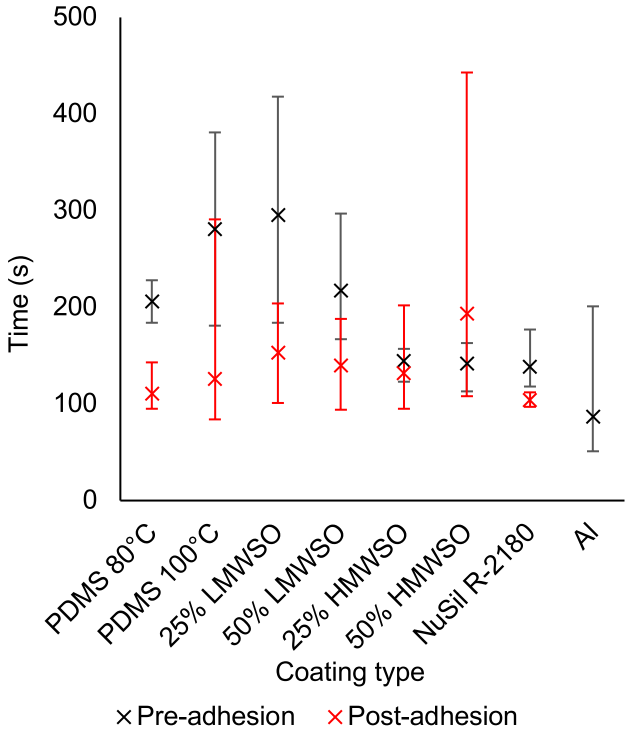

Figures S3. Average freezing time of distilled water droplets on the coatings, with range bars. Measured from start of contact until freezing front reaches top of droplet.

The oil did not have a significant effect on the freezing time, likely a result of the low thermal conductivities of both silicone oil[1, 2] and PDMS[3]. The NuSil R-2180 showed less improvement compared to the PDMS-based coatings, perhaps from minor differences in chemical and thermal properties. The HMWSO coatings had shorter average freezing times than the LMWSO and plain PDMS coatings, which may be explained by the presence of oil on the surface providing more nucleation sites. The coatings predominantly showed a decrease in freezing time after the adhesion testing. Again, this is likely a result of an increase in nucleation sites from damage or dust accumulation. Even with decreases in freezing time after adhesion

testing, all coatings performed better than aluminum, and freezing time is not influenced negatively by oil-infusion long-term.

There were significant instances of supercooling of droplets (cooling of liquid water below 0°C), and many of the droplets on the coated surfaces maintained the liquid state beyond thermal equilibrium. This is a positive result for anti-icing applications, as liquid water droplets require less energy for removal compared to ice.

INDEFINITE NUCLEATION DELAY

An additional finding from these tests was the frequency with which nucleation was delayed indefinitely on the coatings. Indefinite nucleation delay is defined here as delaying nucleation beyond the time for thermal equilibration of the droplets. Supercooling of the droplets below 0°C was anticipated and has been demonstrated as a method of improving overall freezing time, largely via engineered surface textures[4-6]. Indefinite nucleation delay on these surfaces is possibly due to a lack of available nucleation sites on the surfaces.

There was significant supercooling and indefinite nucleation delay on all the coatings. The frequency of indefinite nucleation delay on each coating is plotted in Figure S4. The plain PDMS coatings had exceptional indefinite nucleation delay rates of 75% pre-adhesion testing. All the coatings had more frequent indefinite nucleation delay than aluminum pre-adhesion testing.

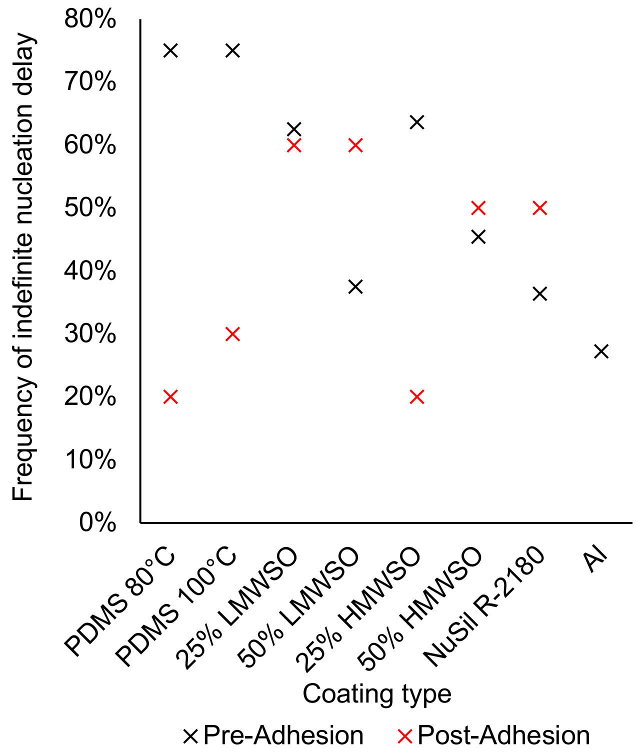

Figure S4. Frequency of indefinite nucleation delay on the surfaces, before and after adhesion testing.

The oil-infused and NuSil R-2180 coatings generally showed smaller changes in frequency of indefinite nucleation between the pre-adhesion and post-adhesion testing, compared to the plain PDMS coatings.

DYNAMIC MECHANICAL ANALYSIS

Dynamic mechanical analysis (DMA) was performed on the polymers to characterize the relative significance of the viscous and elastic components within the mechanical stress response. The glass transition temperature (t_g) for PDMS has been shown to be below −100°C[7-9], though there is also evidence of a secondary peak around −50°C[8, 9], indicating an additional mechanical transition when crystallites begin melting[8-10]. Both temperatures are significantly lower than the test temperature of −10°C.

METHOD

Testing was performed using a TA Instruments Discovery DMA 850 with dual screw film tension fixtures. Samples were cut from films cast in aluminum dishes and had width and thickness of approximately 3 mm and 1.5 mm respectively. The test span was approximately 11 mm. A temperature sweep was run on the samples from −90°C to 30°C with an oscillation of 1 Hz and a temperature ramp rate of 3°C/minute. Three tests were performed for each polymer type and the average results are presented.

RESULTS

Example traces for E', E'' and tanδ are provided in Figure S5. The storage and loss moduli at the relevant test temperatures of 20°C and −10°C were recorded and plotted against the Young's modulus data obtained from compression testing at room temperature (approximately 20°C) in Figure S6. The results show that the storage moduli are much larger than the loss moduli for the polymers and the storage moduli are very similar in value to the Young's moduli. Therefore, it can be argued that the mechanical response is dominated by elastic behavior, and the assumption of Poisson's ratio of 0.5 in the calculation of shear modulus from elastic modulus is appropriate for the temperatures under consideration in this study. The results for PDMS 100°C show good agreement with other studies[8-12].

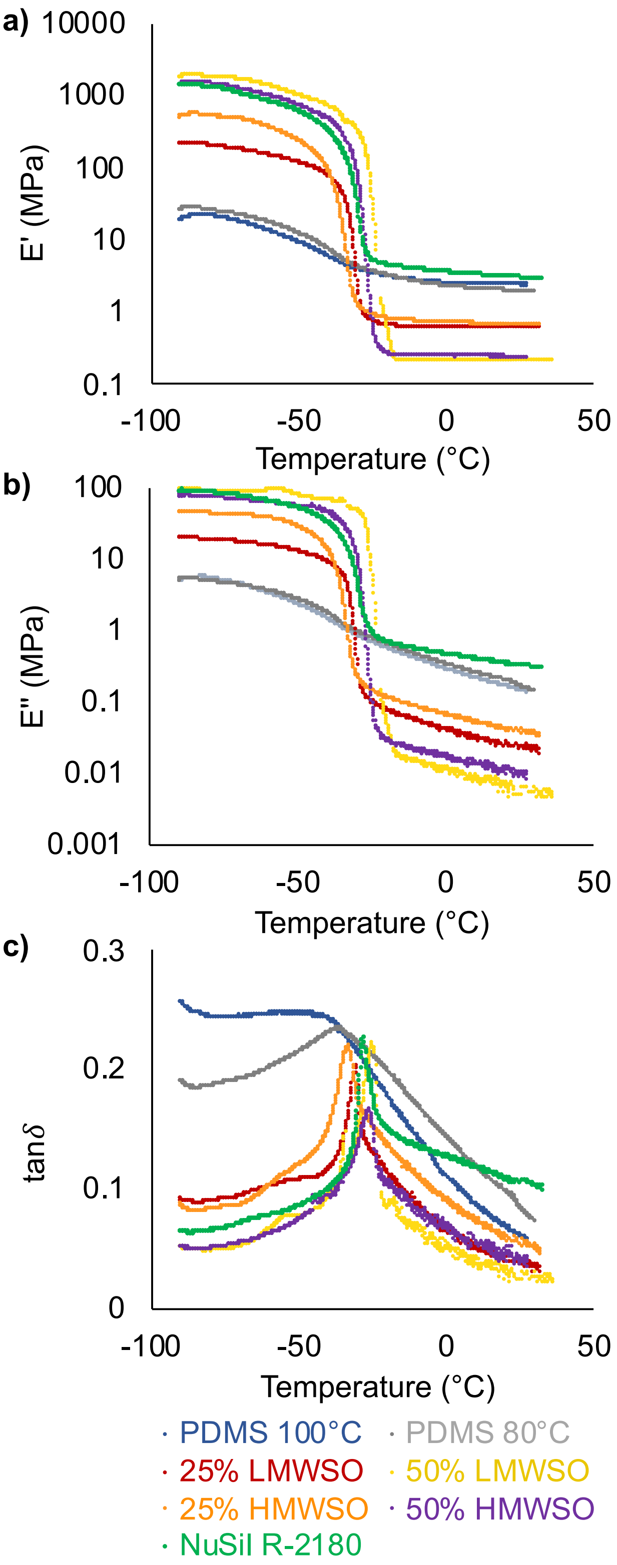

Figure S5. Example traces of a) *E'*, b) *E''* and c) tanδ for the polymer specimens. The secondary transition, denoted by the peak in tanδ, is visible at approximately −50°C for the PDMS 100°C specimen, and between −25°C and −35°C for the other polymers.

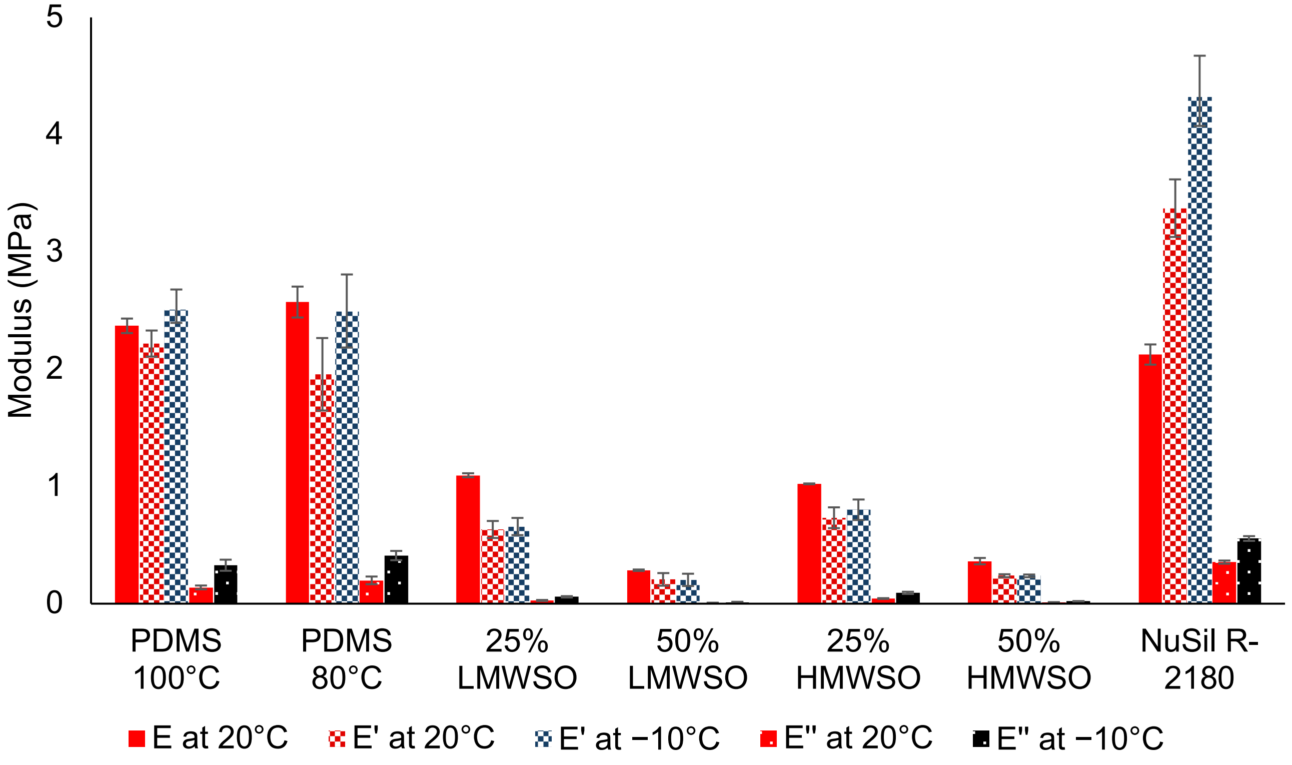

Figure S6. Comparison of Young's modulus (*E*) at 20°C with the storage (*E'*) and loss (*E''*) moduli at 20°C and −10°C for all polymer types. Young's modulus measured via compression testing and loss and storage moduli measured via dynamic mechanical analysis. Results show largely good agreement between the Young's modulus at 20°C and the storage moduli, particularly at −10°C.

In addition to the values for modulus, tanδ values for the polymers at 20°C and −10°C are presented in Figure S7. These results directly show the changing relationship between the storage and loss moduli as temperature of the polymer falls. The lower the tanδ value the more elastic the mechanical response of the polymer. The values of tanδ at −10°C are greater than at 20°C for all the polymer types, which is expected as the t_g of the polymers (and peak tanδ) is much lower. However, at −10°C the storage modulus remains much higher than the loss modulus.

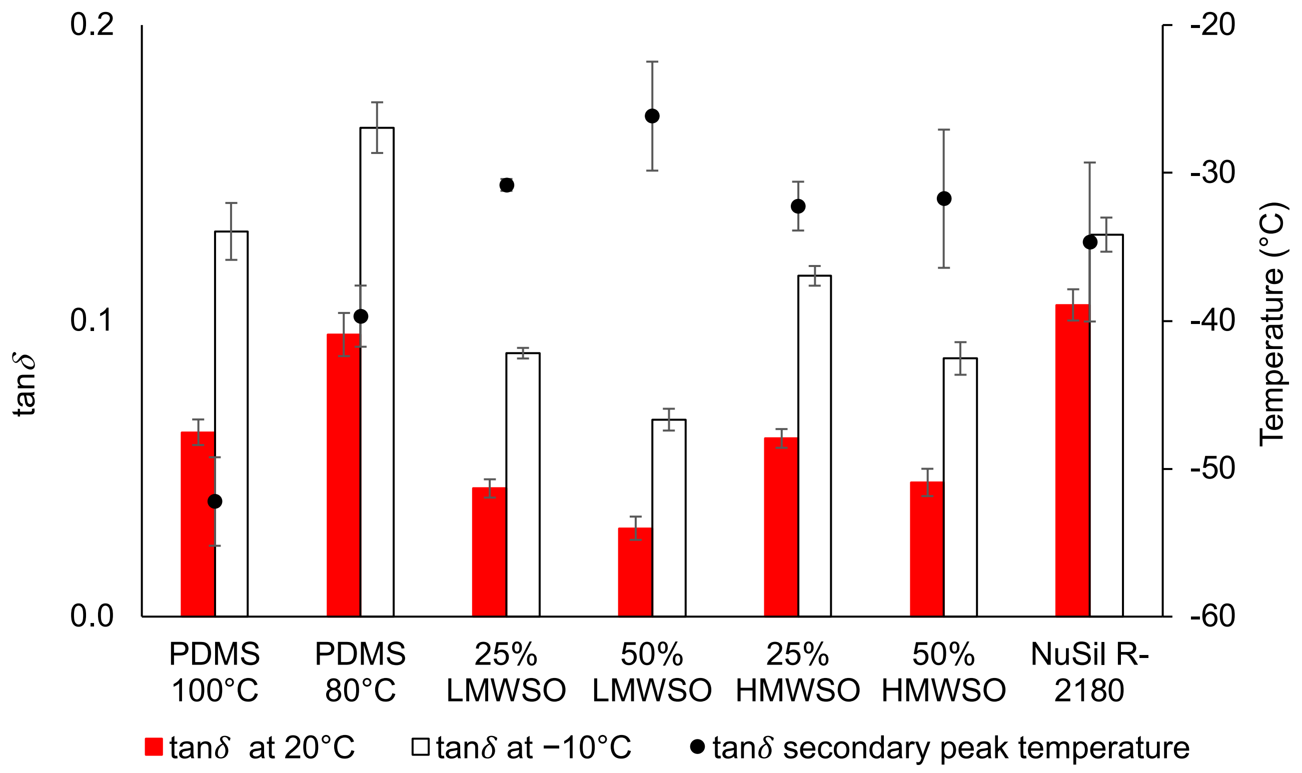

Figure S7. Values of tanδ as measured by dynamic mechanical analysis of the polymers at 20°C and −10°C. The temperatures of the secondary transition (crystallite melting), as denoted by peaks in tanδ, are overlaid.

REFERENCES

(1) Sohn, C. W.; Chen, M. M. Microconvective Thermal Conductivity in Disperse Two-Phase Mixtures as Observed in a Low Velocity Couette Flow Experiment. *Journal of Heat Transfer* **1981**, *103* (1), 47-51. DOI: 10.1115/1.3244428.

(2) Mei, S.; Gao, Y.; Deng, Z.; Liu, J. Thermally Conductive and Highly Electrically Resistive Grease Through Homogeneously Dispersing Liquid Metal Droplets Inside Methyl Silicone Oil. *Journal of Electronic Packaging* **2014**, *136* (1), 011009. DOI: 10.1115/1.4026414.

(3) *SYLGARD™ 184 Silicone Elastomer Kit Technical Data Sheet*. Dow Inc., 2023. https://www.dow.com/en-us/document-

viewer.html?randomVar=6391944654464711505&docPath=/content/dam/dcc/documents/11/11-3184-01-sylgard-184-elastomer.pdf (accessed 7/12/2023).

(4) Tourkine, P.; Le Merrer, M.; Quéré, D. Delayed Freezing on Water Repellent Materials. *Langmuir* **2009**, *25* (13), 7214-7216. DOI: 10.1021/la900929u.

(5) Yue, X.; Liu, W.; Wang, Y. Freezing delay, frost accumulation and droplets condensation properties of micro- or hierarchically-structured silicon surfaces. *International Journal of Heat and Mass Transfer* **2018**, *126* (Part A), 442-451. DOI: https://doi.org/10.1016/j.ijheatmasstransfer.2018.04.165.

(6) Guo, C.; Zhang, M.; Hu, J. Icing delay of sessile water droplets on superhydrophobic titanium alloy surfaces. *Colloids and Surfaces A: Physicochemical and Engineering Aspects* **2021**, *621*, 126587. DOI: https://doi.org/10.1016/j.colsurfa.2021.126587.

(7) Wu, J.; Dan, Q.; Liu, S. Effect of viscoelasticity of PDMS on transfer printing. In *2015 16th International Conference on Electronic Packaging Technology (ICEPT)*, 11-14 Aug. 2015, 2015; pp 759-764. DOI: 10.1109/ICEPT.2015.7236694.

(8) Bosq, N.; Guigo, N.; Persello, J.; Sbirrazzuoli, N. Melt and glass crystallization of PDMS and PDMS silica nanocomposites. *Physical Chemistry Chemical Physics* **2014**, *16* (17), 7830-7840, 10.1039/C4CP00164H. DOI: 10.1039/C4CP00164H.

(9) Liu, Y.; Wang, C.; Jarrell, R. M.; Nair, S.; Wynne, K. J.; Di, D. Icephobic, Pt-Cured, Polydimethylsiloxane Nanocomposite Coatings. *ACS Applied Materials & Interfaces* **2020**, *12* (9), 11180-11189. DOI: 10.1021/acsami.9b20989.

(10) Carlberg, M.; Colombini, D.; Maurer, F. H. J. Ethylene–propylene–rubber (EPR)/polydimethylsiloxane (PDMS) binary polymer blends: Morphology and viscoelastic

properties. *Journal of Applied Polymer Science* **2004**, *94* (5), 2240-2249. DOI: https://doi.org/10.1002/app.21171.

(11) Shi, Y.; Hu, M.; Xing, Y.; Li, Y. Temperature-dependent thermal and mechanical properties of flexible functional PDMS/paraffin composites. *Materials & Design* **2020**, *185*, 108219. DOI: https://doi.org/10.1016/j.matdes.2019.108219.

(12) Wang, C.; Fuller, T.; Zhang, W.; Wynne, K. J. Thickness Dependence of Ice Removal Stress for a Polydimethylsiloxane Nanocomposite: Sylgard 184. *Langmuir* **2014**, *30* (43), 12819-12826. DOI: 10.1021/la5030444.
